# Human phase I metabolism of the novel synthetic cannabinoid 5F-CUMYL-PEGACLONE

**DOI:** 10.1007/s11419-018-0447-4

**Published:** 2018-10-05

**Authors:** Lukas Mogler, Sebastian Halter, Maurice Wilde, Florian Franz, Volker Auwärter

**Affiliations:** 1 Institute of Forensic Medicine, Forensic Toxicology, Medical Center–University of Freiburg, Albertstraße 9, 79104 Freiburg, Germany; 2 Hermann Staudinger Graduate School, University of Freiburg, Hebelstraße 27, 79104 Freiburg, Germany; 3 Faculty of Medicine, University of Freiburg, Breisacherstrasse 153, 79110 Freiburg, Germany

**Keywords:** 5F-CUMYL-PEGACLONE, Metabolism in vivo and in vitro, *γ*-Carbolinone, Human liver microsomes, Urine analysis, 5F-SGT-151

## Abstract

**Purpose:**

5F-CUMYL-PEGACLONE is a recently emerged *γ*-carbolinone derived synthetic cannabinoid. The present study aimed to identify phase I metabolites to reliably prove consumption of the substance by urine analysis and to differentiate from the uptake of the non-fluorinated analog CUMYL-PEGACLONE.

**Methods:**

For metabolite characterization, phase I metabolites were analyzed by liquid chromatography–high resolution mass spectrometry after incubation with pooled human liver microsomes. Reliability of the biomarkers was evaluated by analysis of human urine samples (*n *= 20) by liquid chromatography–triple quadrupole tandem mass spectrometry. Sample preparation included *β*-glucuronidase treatment followed by liquid-liquid extraction.

**Results:**

In total, 15 metabolites were detected in vivo and characterized. Metabolic reactions were primarily observed at the *γ*-carbolinone core and the 5-fluoropentyl chain, and included *N*-dealkylation, hydroxylation, hydrolytic defluorination, formation of a dihydrodiol, oxidation to the pentanoic acid metabolite and formation of the propionic acid metabolite. Six of these metabolites were identical with phase I metabolites of CUMYL-PEGACLONE, which must be considered for interpretation of analytical findings in urine samples.

**Conclusions:**

5F-CUMYL-PEGACLONE was subject to extensive metabolism in humans. The propionic acid metabolite was the most abundant metabolite in all urine samples and should be targeted when maximum sensitivity is needed (e.g., drug abstinence control). However, this metabolite also occurs in the biotransformation of the non-fluorinated analog and is, therefore, not a compound-specific marker. For differentiation, a metabolite hydroxylated at the *γ*-carbolinone core showed to be the most reliable marker and should be used as an additional target analyte.

**Electronic supplementary material:**

The online version of this article (10.1007/s11419-018-0447-4) contains supplementary material, which is available to authorized users.

## Introduction

**Fig. 1:**
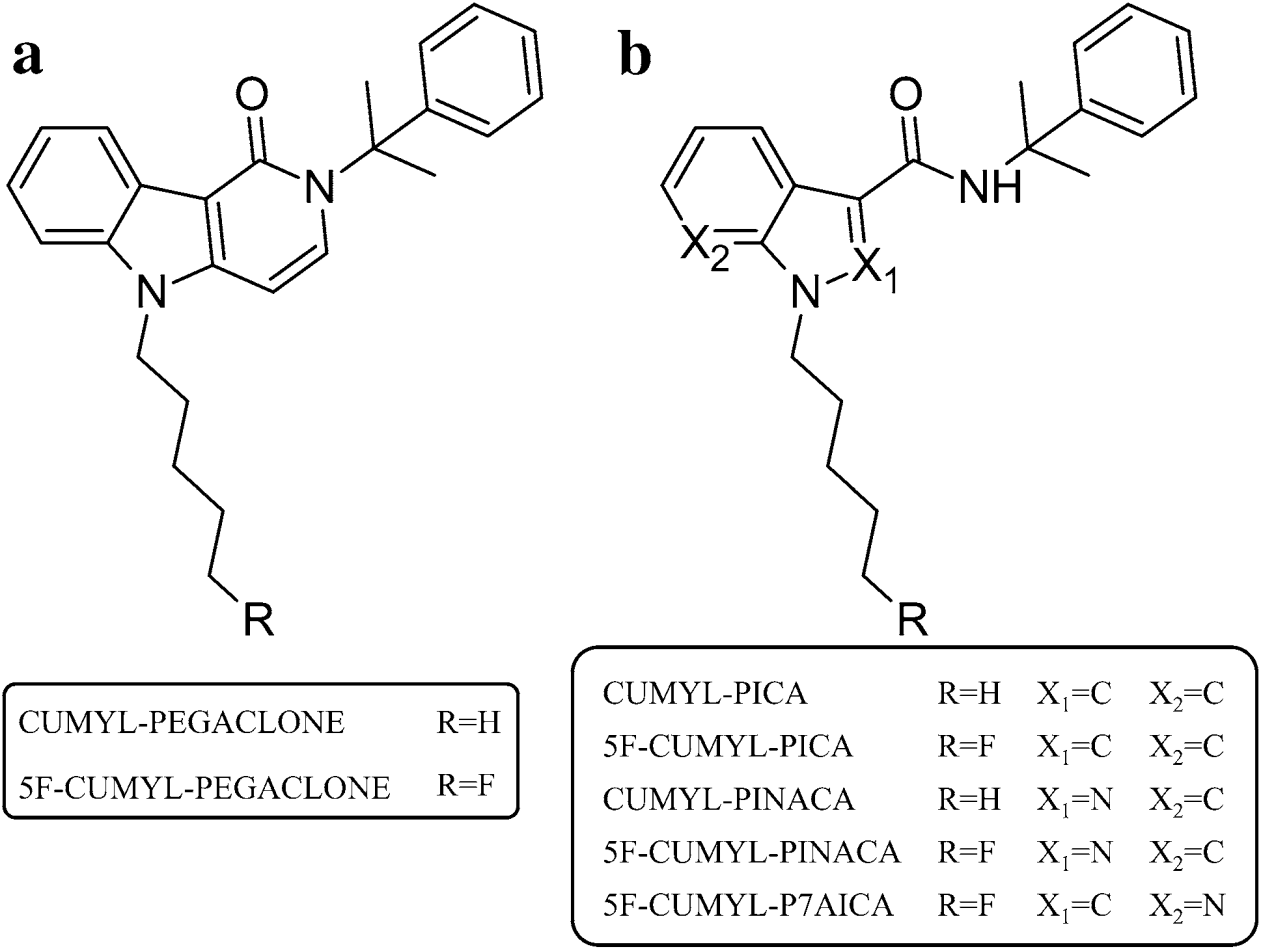
Structures of **a***γ*-carbolinone derived cumyl synthetic cannabinoids (SCs),  and **b** indole, indazole or azaindole derived cumyl carboxamide type SCs

Synthetic cannabinoids (SCs) are a class of designer drugs recreationally used to mimic the effects of cannabis. Among the new psychoactive substances (NPS), SCs are still among the largest subgroups monitored by the European Monitoring Centre for Drugs and Drug Addiction (EMCDDA). According to recent reports from July 2018, there were 179 different SCs on the European drug market [1]. SCs were detected in “legal high” products in 2008 for the first time [2, 3]. Since then, clandestine laboratories synthesized compounds often based on pharmaceutical research papers and patent applications [4, 5]. For instance, several highly potent SCs that emerged on the drug market were first described in a patent application of Bowden and Williamson (“SGT-compounds”) [6]. These drugs are characterized by a cumyl substituent, which is attached to indole, indazole or azaindole core structures by a linker moiety (Fig. 1). Pharmacological evaluation of some of these substances showed that cumyl derived SCs are potent agonists at the cannabinoid receptors CB_1_ and CB_2_ [7, 8]. The first cumyl-carrying SC comprising a *y*-carbolinone core structure occurred on the German drug market in December 2016. CUMYL PEGACLONE (5-pentyl-2-(2-phenylpropan-2-yl)-2,5-dihydro-1*H*-pyrido[4,3-*b*]indol-1-one) (Fig. 1) was identified in an herbal mixture and was also sold under the street name SGT-151. The compound showed full agonistic properties with binding affinities in the low nanomolar range at CB_1_ and CB_2_ [9, 10]. The human phase I metabolism of CUMYL-PEGACLONE was recently described [10, 11]. In 2018, a substance purchased as a “research chemical” online was identified as 5F-CUMYL-PEGACLONE (5-(5-fluoropentyl)-2-(1-methyl-1-phenylethyl)-1*H*-pyrido[4,3-*b*]indol-1-one) (Fig. 1) in our laboratory. Exchange of a hydrogen atom of the *N*-pentyl side chain by a fluorine atom at C_5_ has been a common modification also applied to other core structures by clandestine chemists [12, 13].

To prove the uptake of SCs, urine is often the preferred biological matrix for forensic and clinical toxicology, particularly in drug abstinence testing when longer detection windows are needed. In urine, metabolites of SCs are the target compounds as parent SCs are rarely detectable due to extensive metabolism. To enhance sensitivity, cleavage of phase II conjugates is a common step prior to liquid chromatography–mass spectrometry (LC–MS) analysis [14, 15]. The major problem in urine analysis of SC metabolites is the lack of commercially available reference standards. To circumvent this problem, in vitro generation of phase I metabolites, e.g., by human liver microsomes (HLMs), human hepatocytes, or even fungi was established [16–18]. Tentative identification and characterization of the metabolites are commonly carried out by high resolution mass spectrometry (HRMS) techniques that allow identification based on accurate mass and isotopic patterns. For metabolite screening in urine, liquid chromatography–tandem mass spectrometry (LC–MS/MS) methods are still a frequently used alternative to HRMS in forensic and clinical toxicology when maximum sensitivity for routine purposes is required [19].

The aim of the present study was to tentatively identify and characterize human phase I metabolites produced in an HLM assay by liquid chromatography–quadrupole time-of-flight mass spectrometry (LC–QToF-MS). These data were compared with the phase I metabolite profile detected in urine samples from drug users by an LC–MS/MS screening method where at least two metabolites were detected. Based on these results, phase I metabolites were evaluated as targets for urine screening to reliably prove 5F-CUMYL-PEGACLONE consumption and to differentiate from the uptake of the non-fluorinated analog CUMYL-PEGACLONE.

## Materials and methods

### Chemicals and reagents

Formic acid (Rotipuran^®^ ≥ 98%, p.a.) and potassium hydrogen phosphate (≥ 99%, p.a.) were obtained from Carl Roth (Karlsruhe, Germany); acetonitrile (ACN) (LC–MS grade), ammonium formate 10 M (99.995%), potassium hydroxide [puriss. p.a. ≥ 86% (T) pellets] and superoxide dismutase (SOD) (≥ 3000 units/mg protein from bovine erythrocytes) from Sigma-Aldrich (Steinheim, Germany); pooled HLMs (50 donors, 20 mg/mL protein in 250 mM sucrose), NADPH regenerating solutions A and B (reductase activity 0.43 µmol/min/mL), and potassium phosphate buffer 0.5 M (pH 7.5) from Corning (Corning, NY, USA); *β*-glucuronidase (*E. coli* K 12) from Roche Diagnostics (Mannheim, Germany). Mobile phase A (1% v/v ACN, 0.1% v/v HCOOH, 2 mM ammonium formate in water) and mobile phase B (0.1% v/v HCOOH, 2 mM ammonium formate in ACN) were freshly prepared prior to analysis. 5F-CUMYL-PEGACLONE and CUMYL-PEGACLONE reference standards were obtained from Chiron AS (Trondheim, Norway). Stock solutions (1 mg/mL) were prepared in ACN and stored at −20 °C until analysis.

### Pooled human liver microsome assay

Total assay volume of 100 µL consisted of 5 µL pooled HLM solution, 1 µL parent compound stock solution (1 mg/mL in ACN), 5 µL NADPH regenerating solution A, 1 µL NADPH regenerating solution B, 10 µL SOD, 20 µL phosphate buffer, and 58 µL deionized water. Incubation was conducted for 30 min at 37 °C. The incubation was quenched by adding 100 µL of ice-cold ACN. After centrifugation, the supernatant was transferred into a separate vial and stored at −20 °C. Two negative control samples were processed in the same way. One was performed with 5 µL phosphate buffer instead of pooled HLMs and a second with 1 µL ACN instead of the substrate. Prior to LC–MS/MS analysis the supernatant was diluted 1:10 in mobile phase A/B (70:30, v/v). For LC–QToF-MS analysis in the electrospray ionization (ESI) mode, 100 µL supernatant was evaporated until dryness and reconstituted in 25 µL mobile phase A/B (70:30, v/v).

### Human urine samples

The urine samples (*n *= 20) were collected during LC–MS/MS routine screening for metabolites of synthetic cannabinoids between April 2018 and July 2018. Samples were rated positive for the consumption of an SC when at least two metabolites were detected, which met the identification criteria for LC–MS/MS analyses as defined by the German Society of Toxicological and Forensic Chemistry (GTFCh) [19]. Incubations of pooled HLMs and one urine sample (with a corresponding blood sample proving the consumption of 5F-CUMYL-PEGACLONE) served as a positive control. Urine samples positive for CUMYL-PEGACLONE metabolites (*n *= 6) were also used in the previous metabolism study [11]. All urine samples were sent to the laboratory of forensic toxicology in Freiburg (Germany) for drug abstinence control testing and all analyses were conducted in accordance with the inquiry of the respective client.

### Urine sample preparation

An aliquot of 0.5 mL of urine was treated with 0.5 mL phosphate buffer (pH 6) and 30 µL *β*-glucuronidase for conjugate cleavage at 45 °C for 60 min. Liquid-liquid extraction was performed by adding 1.5 mL ACN and 0.5 mL of a 10 M ammonium formate solution. After shaking and centrifugation the organic layer was transferred into a separate vial and evaporated to dryness under a nitrogen stream at 40 °C. Reconstitution was done in 200 µL mobile phase A/B (70:30, v/v) prior to LC–MS/MS analysis or in 25 µL mobile phase A/B (70:30, v/v) prior to LC–QToF-MS analysis.

### LC–ESI-QToF-MS experiments

LC–ESI-QToF-MS analysis was performed on an impact II™ QToF instrument coupled with an Elute HPLC system (both from Bruker Daltonik, Bremen, Germany). Chromatographic separation was achieved on a Kinetex^®^ C18 column (2.6 µm, 100 Å, 100 × 2.1 mm; Phenomenex, Aschaffenburg, Germany), protected by an equivalent Security Guard™ ULTRA catridge precolumn (Phenomenex), applying gradient elution as follows: total LC run time was 15 min with a mobile phase B starting concentration of 30%, linearly increased to 45% in 9.0 min, further increased to 70% in 1.0 min, further increased to 95% in 1.0 min, held for 2.0 min, decreased to starting conditions of 30% in 0.1 min and held for 1.9 min for re-equilibration. The flow rate was set to 0.4 mL/min. The autosampler was cooled down to 10 °C. Column oven temperature was 40 °C. The injection volume was 10 µL. HyStar™ version 3.2 and DataAnalysis version 4.2 (both from Bruker Daltonik) were used for data acquisition and processing, respectively. The QToF-MS was operated in positive ionization mode acquiring spectra in the range of *m/z* 30–600 in full scan (acquisition rate of 4.0 Hz), and broadband collision induced dissociation (bbCID) data were acquired in one run. The collision energy applied for bbCID was 30 ± 6 eV. Instrument parameters were set as described previously [13]. CID fragmentation experiments for 5F-CUMYL-PEGACLONE were performed with the reference standard solution at 1 µg/mL with LC–HRMS parameters as stated earlier.

### LC–ESI–MS/MS experiments

LC–ESI-QTRAP-MS analysis was performed with a Nexera X2 UHPLC (Shimadzu, Duisburg, Germany) coupled to a QTRAP^®^ 5500 triple quadrupole linear ion trap instrument (SCIEX, Darmstadt, Germany). Chromatographic parameters, injection volume, autosampler and column oven temperature were as described earlier.

The QTRAP-MS was operated with positive ionization in multiple reaction monitoring (MRM) mode and enhanced product ion (EPI) scan mode. The respective potentials (declustering potential (DP), entrance potential (EP), collision energies (CE), and collision cell exit potential (CXP) of the multiple reaction monitoring (MRM) ion transitions of the parent compound were optimized under direct infusion (10 ng/mL) (see Supplementary Material Table S1).

### In vitro metabolite identification and characterization

Metabolites generated in the pooled HLM assay were tentatively identified and characterized by LC–QToF-MS in manual data processing with following criteria: MS peak area > 1 × 10^5^ cps, mass error of the precursor ion < 5 ppm, signal-to-noise ratio > 3:1, and mass tolerance for fragment ions ± 10 ppm. First of all, a list of hypothetical metabolites was generated on the basis of previous metabolism studies of structurally related SCs [11, 20, 21]. To avoid missing the main metabolites, precursor ions were searched via typical fragment ions in the bbCID data. Based on the LC–HRMS data, an LC–MS/MS MRM-method was developed, comprising the two most abundant ion transitions of each metabolite applying the optimized MS parameters of the parent compound (see Supplementary Material Table S2). The most abundant fragment ions were identified by recording EPI spectra of the metabolites. For EPI scans, EP was set to 10 V and a CE of 35 V with a spread of ± 15 V was applied. The two most abundant in vitro metabolites (M10 and M12) were integrated into an existing LC–MS/MS routine screening method to screen for positive urine samples.

### Identification of metabolites in urine samples and biomarker evaluation

A metabolite was regarded as “identified” in LC–MS/MS analysis of urine samples when the following criteria were met: signal-to-noise ratio > 3:1; peak area > 1 × 10^4^ cps; retention time (RT) (± 0.1 min); matching EPI spectra (when in vitro reference spectra where available); metabolites, not detectable in the pooled HLM assay, but additionally confirmed by accurate mass (LC-QToF-MS analysis). In order to compare the relative abundances of the metabolites as a rough estimate of their concentrations, peak area ratios were calculated by dividing the peak area of a detected metabolite in a sample by the peak area of the most abundant metabolite (M06) in the same sample. In this way, the metabolites were ranked by their mean peak area ratios over all analyzed urine samples. The relative standard deviations (RSDs) of the mean peak area ratios provide an estimation of the variability of the metabolites’ rank positions within the investigated sample collective.

## Results and discussion

### LC–ESI-QToF-MS characterization of 5F-CUMYL-PEGACLONE

**Fig. 2:**
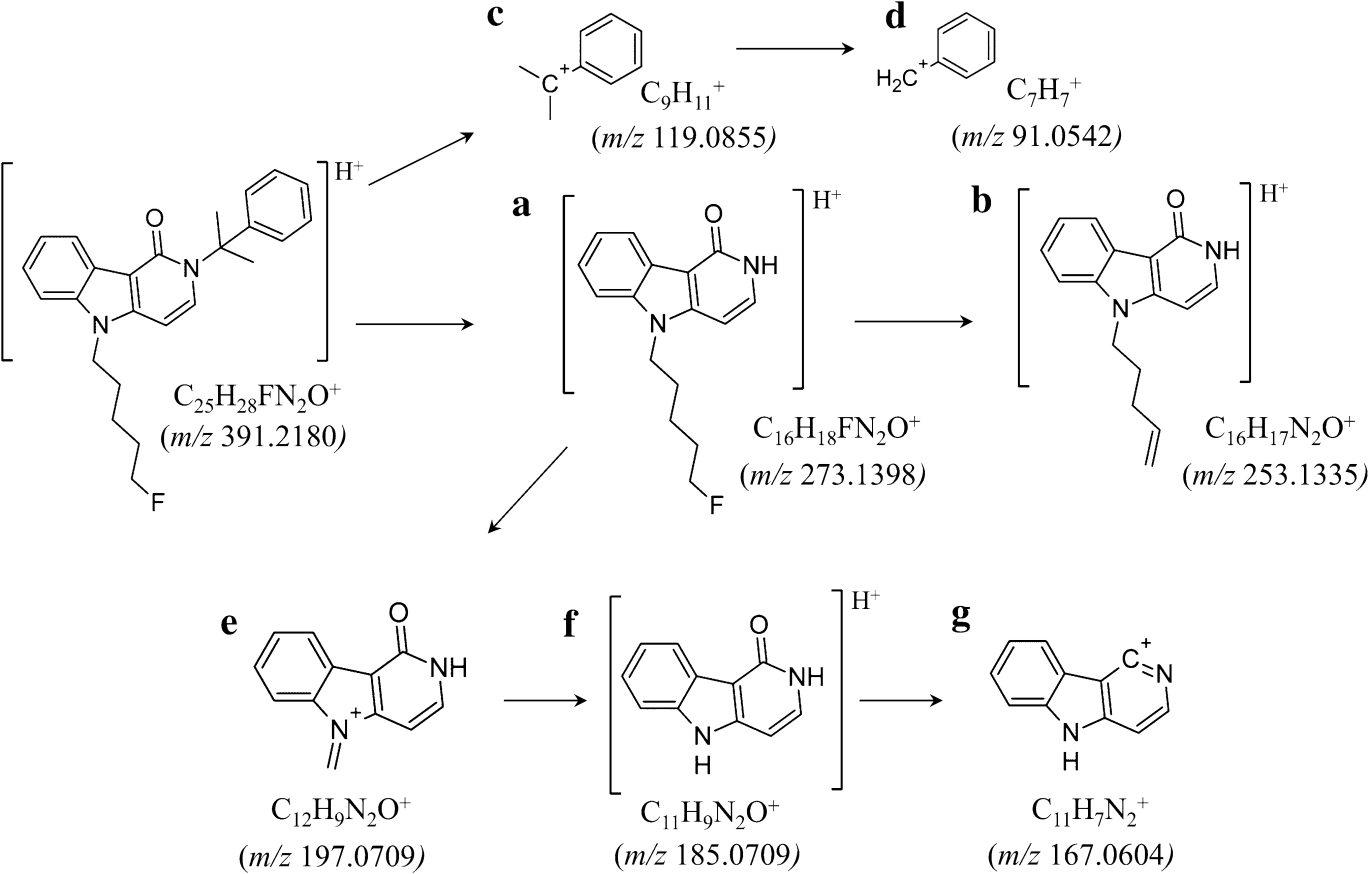
Proposed pathways for the formation of the main characteristic fragment ions in the liquid chmromatography–electrospray ionization-collision induced dissociation spectra of 5F-CUMYL-PEGACLONE

To investigate the ESI-MS fragmentation behavior of 5F-CUMYL-PEGACLONE (C_25_H_28_FN_2_O^+^; *m/z* 391.2180), a solution of 1 µg/mL was analyzed by LC–ESI-QToF-MS in full scan and bbCID mode. The proposed fragmentation pathways of 5F-CUMYL-PEGACLONE are shown in Fig. 2. CID fragmentation led to the main fragment ion a (C_16_H_18_FN_2_O^+^; *m/z* 273.1398), most probably due to α-cleavage between the lactam nitrogen of the core system and the benzyl carbon of the cumyl moiety (Fig. 2a). Fragment b (C_16_H_17_N_2_O^+^; *m/z* 253.1335) is the product of HF elimination of fragment a (Fig. 2b). This pathway has previously been described for SCs with an indazole core ring [22]. Fragmentation of the cumyl moiety leads to the dimethylbenzyl ion c (C_9_H_11_^+^; *m/z* 119.0855) (Fig. 2c), which is further degraded by twofold loss of CH_2_ to the tropylium ion d (C_7_H_7_^+^; *m/z* 91.0542). A characteristic fragmentation pathway for the *γ*-carbolinone core is formation of the three fragment ions e, f and g by further fragmentation of fragment ion a (Fig. 2e, f, g). Fragment ion f (C_11_H_9_N_2_O^+^; *m/z* 185.0709) is the most prominent fragment of those and represents the *γ*-carbolinone ion. Fragment g (C_11_H_7_N_2_^+^; *m/z* 167.0604) is most probably formed by the loss of H_2_O from fragment ion f. Comparable degradations of SCs based on tricyclic core systems have been described for carbazole derived SCs [13]. Fragment ion e (C_12_H_9_N_2_O^+^; *m/z* 197.0709) is less produced by CID and could be referred to a dealkylation of fragment ion a in allylic position leading to a *γ*-carbolinone-*N*-methyl ion. This assumption is supported by the fact that fragment ion e was not detectable in CID spectra of *N*-dealkylated metabolites. The corresponding spectra can be found in Supplementary Material Fig. S1.

### Microsomal phase I metabolism of 5F-CUMYL-PEGACLONE

In the pooled HLM assay, 30 phase I metabolites were generated and characterized. None of the metabolites were detected in the control samples. The observed metabolic reactions in vitro were hydroxylation, hydrolytic defluorination, *N*-dealkylation, aldehyde/ketone formation, formation of a dihydrodiol, oxidation to the carboxylic acid and combinations thereof. A detailed list of the detected in vitro metabolites can be found in the Supplementary Material (Table S3). To update an existing LC–MS/MS screening method, the ion transitions of the most abundant in vitro metabolites (in vivo confirmed as M10 and M12) were integrated. Subsequently, 20 urine samples were found positive for these metabolites, which met the set identification criteria [19].

### Human in vivo phase I metabolites of 5F-CUMYL-PEGACLONE

In total, 15 different phase I metabolites were detected in the investigated set of urine samples. The metabolic reactions in vivo included hydroxylation, formation of a dihydrodiol, hydrolytic defluorination, *N*-dealkylation, oxidation to the pentanoic acid metabolite, side chain degradation to the propionic acid metabolite and combinations thereof. The metabolites were named serially numbered in the order of their retention times (RTs). The parent compound was not detected in any of the urine samples. Since the identification of the particular position of functional groups introduced by metabolic reactions would require isolation of metabolites for structure elucidation, e.g., by nuclear magnetic resonance (NMR) spectroscopy or synthesis of reference material, the exact chemical structures of some metabolites remain unclear. Table 1 shows the detected in vivo phase I metabolites with the proposed biotransformation step, tentative localizations of the metabolic modifications, detected [M+H]^+^ precursor masses including their mass errors, elemental compositions, characteristic fragment ions including their mass errors and the overall rank position. The product ion mass spectra for localization of metabolic modification are shown in Supplementary Material Table S4. Accurate masses were obtained from signals of the in vitro assay.

**Table 1:** Human phase I metabolites of 5F-CUMYL-PEGACLONE detected from 20 urine samples

ID	RT (min)	Biotransformation	Location	Ranking position	Mean area ratio (%)	RSD (%)	Calculated [M+H]^+^	Ion formula	Mass error (ppm)	Diagnostic product ions calc. (*m/z*)	Diagnostic product ions formula	Diagnostic product ions mass error (ppm)
M01^a^	2.6	*N*-Dealkylation + monohydroxylation	CBL, 5F-P	8	0.67	219	319.1441	C_20_H_19_N_2_O_2_	0.3	201.0659	C_11_H_9_N_2_O_2_	0.9
										119.0855	C_9_H_11_	0.2
M02^a^	3.1	*N*-Dealkylation + monohydroxylation	CBL, 5F-P	13	0.32	348	319.1441	C_20_H_19_N_2_O_2_	−0.5	201.0659	C_11_H_9_N_2_O_2_	−1.8
										119.0855	C_9_H_11_	−3.8
M03	3.5	Dihydroxylation	CBL, 5F-P	10	0.12	235	423.2078	C_25_H_28_FN_2_O_3_	−0.6	305.1296	C_16_H_18_FN_2_O_3_	0.9
										213.0659	C_12_H_9_N_2_O_2_	0.6
M04	3.6	Hydrolytic defluorination + monohydroxylation	CBL, 5F-P	6	1.04	140	405.2173	C_25_H_29_N_2_O_3_	−0.1	287.1390	C_16_H_19_N_2_O_3_	0.3
										213.0659	C_12_H_9_N_2_O_2_	0.6
M05	3.8	Dihydroxylation	CBL, 5F-P	14	0.41	286	423.2078	C_25_H_28_FN_2_O_3_	−0.9	305.1296	C_16_H_18_FN_2_O_3_	0.3
										213.0659	C_12_H_9_N_2_O_2_	−2
M06^a^	4.4	Propionic acid	5F-P	1	100	0	375.1703	C_23_H_23_N_2_O_3_	−0.7	257.0923	C_14_H_13_N_2_O_3_	−0.9
										197.0709	C_12_H_9_N_2_O	−0.7
M07	4.7	Hydrolytic defluorination + monohydroxylation	CBL, 5F-P	7	0.56	162	405.2173	C_25_H_29_N_2_O_3_	−0.1	287.1390	C_16_H_19_N_2_O_3_	0
										213.0659	C_12_H_9_N_2_O_2_	−0.3
M08	6.0	Pentanoic acid	5F-P	2	6.53	123	403.2016	C_25_H_27_N_2_O_3_	−0.1	285.1234	C_16_H_17_N_2_O_3_	−0.4
										267.1128	C_16_H_15_N_2_O_2_	−0.7
M09	6.0	Dihydrodiol formation	CBL	11	0.17	241	425.2235	C_25_H_30_FN_2_O_3_	−0.4	307.1452	C_16_H_20_FN_2_O_3_	−0.5
										197.0709	C_12_H_9_N_2_O	−0.4
M10	6.3	Hydrolytic defluoration	5F-P	12	0.04	353	389.2224	C_25_H_29_N_2_O_2_	0.5	271.1441	C_16_H_19_N_2_O_2_	0.3
										197.0709	C_12_H_9_N_2_O	0
M11	6.5	Monohydroxylation	5F-P	9	0.16	184	407.2129	C_25_H_28_FN_2_O_2_	0.1	289.1335	C_19_H_17_N_2_O	−3.4
										197.0709	C_12_H_9_N_2_O	0
M12	7.1	Monohydroxylation	5F-P	4	3.05	122	407.2129	C_25_H_28_FN_2_O_2_	−0.4	289.1335	C_19_H_17_N_2_O	−4.8
										197.0709	C_12_H_9_N_2_O	−0.3
M13	7.8	Monohydroxylation	CBL	3	4.83	154	407.2129	C_25_H_28_FN_2_O_2_	−0.8	289.1335	C_16_H_18_FN_2_O_2_	−0.5
										201.0659	C_11_H_9_N_2_O_2_	0.4
M14	8.6	Monohydroxylation	CUM	15	0.01	202	407.2129	C_25_H_28_FN_2_O_2_	−0.8	273.1398	C_16_H_18_FN_2_O	0.2
										135.1168	C_9_H_11_O	3.5
M15	9.6	Monohydroxylation	CBL	5	3.02	165	407.2129	C_25_H_28_FN_2_O_2_	−1	289.1335	C_16_H_18_FN_2_O_2_	−0.3
										201.0659	C_11_H_9_N_2_O_2_	−0.4

High-resolution mass spectrometric data are shown from a urine sample

*RSD* relative standard deviation, *RT* retention time, *CUM* cumyl moiety, *5F*-*P* 5-fluoropentyl chain, *CBL γ*-carbolinone core system

^a^Not detected in vitro

The metabolites M11–M15 with [M+H]^+^ at *m/z* 407.2129 are formed by hydroxylation within human phase I metabolism. M11 and M12 are monohydroxylated at the 5-fluoropentyl chain with the characteristic product ions at *m/z* 289.1347, 197.0709 and unaltered cumyl moiety fragments c and d. The hydroxy group of M13 and M15 could be located at the *γ*-carbolinone core, indicated by the fragment ion at *m/z* 289.1347 in combination with the diagnostic core fragments at *m/z* 201.0659 and 183.0553. M14 showed the fragment ions at *m/z* 273.1398 135.11686, suggesting a hydroxylated cumyl moiety.

Further hydroxylation led to the dihydroxylated metabolites M03 and M05 with [M+H]^+^ at *m/z* 423.2078. Both metabolites are hydroxylated at the core system and the 5-fluoropentyl chain yielding in diagnostic product ions at *m/z* 305.1296, 213.0659, and unaltered cumyl moiety fragment c.

Monohydroxylation in combination with *N*-dealkylation of the 5-fluoropentyl chain led to the metabolites M01 and M02 ([M+H]^+^ at *m/z* 319.1441). The hydroxylation occurs at the *γ*-carbolinone core with fragment ions at *m/z* 201.0659 and 183.0553.

M10 is formed by hydrolytic defluorination with [M+H]^+^ at *m/z* 389.2224 and diagnostic product ions at 271.1441, with unaltered fragment ions c and d for the cumyl moiety as well as unaltered fragment ions e and f for the *γ*-carbolinone core. Oxidation of M10 most likely led to the pentanoic acid metabolite M08 with [M+H]^+^ at *m/z* 403.2016. The diagnostic product ion at *m/z* 285.1234 followed by elimination of CO_2_ (fragment ion at *m/z* 239.1179) exclude that this metabolite is formed by ketone formation in combination with hydroxylation, which was described as a major metabolite for CUMYL-PEGACLONE [11].

Oxidative degradation of the 5-fluoropentyl chain led to the propionic acid metabolite of M08 with [M+H]^+^ at *m/z* 375.1703 (M06). The characteristic fragment ion at *m/z* 257.0921 and the unaltered fragment ions c and d for the cumyl moiety as well as unaltered fragment ion e for the *γ*-carbolinone core were detected. M06 was not detected in the pooled HLM assay, but turned out to be the most abundant metabolite of 5F-CUMYL-PEGACLONE in human phase I metabolism under the chosen analytical conditions. A similar biotransformation producing a highly abundant propionic acid metabolite has already been described for the SC 5F-MDMB-PICA [15].

Monohydroxylation in combination with hydrolytic defluorination generated M04 and M07 with [M+H]^+^ at *m/z* 405.2173 and a characteristic product ion at m/z 287.1390. The hydroxylation site was the *γ*-carbolinone core with fragment ions at *m/z* 201.0659 and 183.0553.

The metabolite M09 with [M+H]^+^ at *m/z* 425.2235 could be referred to the formation of a dihydrodiol functionality at the *γ*-carbolinone core with diagnostic product ions at *m/z* 307.1452 and the unaltered cumyl fragment c most probably formed by hydrolysis after epoxidation.

**Fig. 3:**
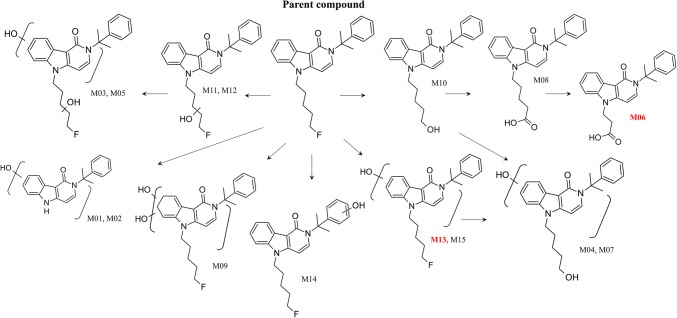
Postulated human phase I biotransformation pathways of 5F-CUMYL-PEGACLONE for 20 urine samples investigated. Main metabolites suggested as analytical targets for urine analysis are highlighted in red (M06 and M13)

The postulated phase I metabolic pathways of 5F-CUMYL-PEGACLONE in humans are shown in Fig. 3.

### Comparison of in vivo and in vitro results

Twelve of the detected in vivo metabolites could be confirmed by corresponding signals in pooled HLM samples. The in vivo metabolites M01 and M02, both *N*-dealkylated and monohydroxylated at the core system, could not be detected in the HLM assay. This might be explained by a relatively weak tendency of HLMs to perform multiple biotransformations under the chosen conditions. The most abundant in vivo metabolite was the propionic acid metabolite. This metabolite is most probably formed by *β*-oxidation of the pentanoic acid metabolite. This metabolite was not identified in the HLM assay because *β*-oxidation mainly occurs in mitochondria, which are not part of the microsomal fraction used in this assay. However, its presence in urine was revealed by the bbCID scan approach for unexpected metabolites, and further characterization (accurate masses and fragmentation pattern) was performed by LC–QToF-MS analysis. A general limitation of pooled HLM assays can be seen in the fact that they do not produce the full human phase I (and II) metabolite spectrum and differ from other in vitro models like hepatocytes [17]. In fact, urine samples (which were available in this study) provide the only valid way for evaluation and confirmation of human SC metabolites suitable as urinary biomarkers—whether predicted by reference spectra of liver microsome assays or by other means [13].

### Comparison with metabolic pathways of CUMYL-PEGACLONE

To detect identical metabolites with the non-fluorinated analog CUMYL-PEGACLONE, a set of six urine samples only positive for metabolites of CUMYL-PEGACLONE was analyzed by the LC–MS/MS method described above. Additionally, the metabolite ranking was compared to the data of the human metabolism study of CUMYL-PEGACLONE [11]. Within this comparative analysis, six identical metabolites (M01, M02, M04, M06, M08, M10) were detected. An overlayed chromatogram is shown in the Supplementary Material (Fig. S2).

### Evaluation of phase I metabolites as consumption markers

To evaluate the identified in vivo metabolites as reliable consumption markers, a qualitative ranking of the metabolites by their peak areas was conducted.

In the investigated collected urine samples, M06 was the most abundant metabolite in each of the urine samples. Thus, this main in vivo phase I metabolite can serve as a highly sensitive marker for 5F-CUMYL-PEGACLONE consumption. Because this metabolite was also formed in the metabolism of the non-fluorinated analog (rank position 5 out of 22), selective metabolites must be included in screening methods for unambiguous identification of the consumed compound [11].

M06 is most likely generated by *β*-oxidation or oxidative degradation of the 5-fluoropentyl chain. Previous biotransformation steps should include hydrolytic defluorination (M10) and oxidation to the pentanoic acid metabolite (M08). The potential intermediate from oxidative degradation, a butanoic acid metabolite ([M+H]^+^ at *m/z* 389.1860), could not be detected in the LC–HRMS bbCID approach. In contrast, for AM-2201, which also carries a 5-fluoropentyl side chain, the butanoic acid metabolite was suggested as a main metabolite [12].

**Fig. 4:**
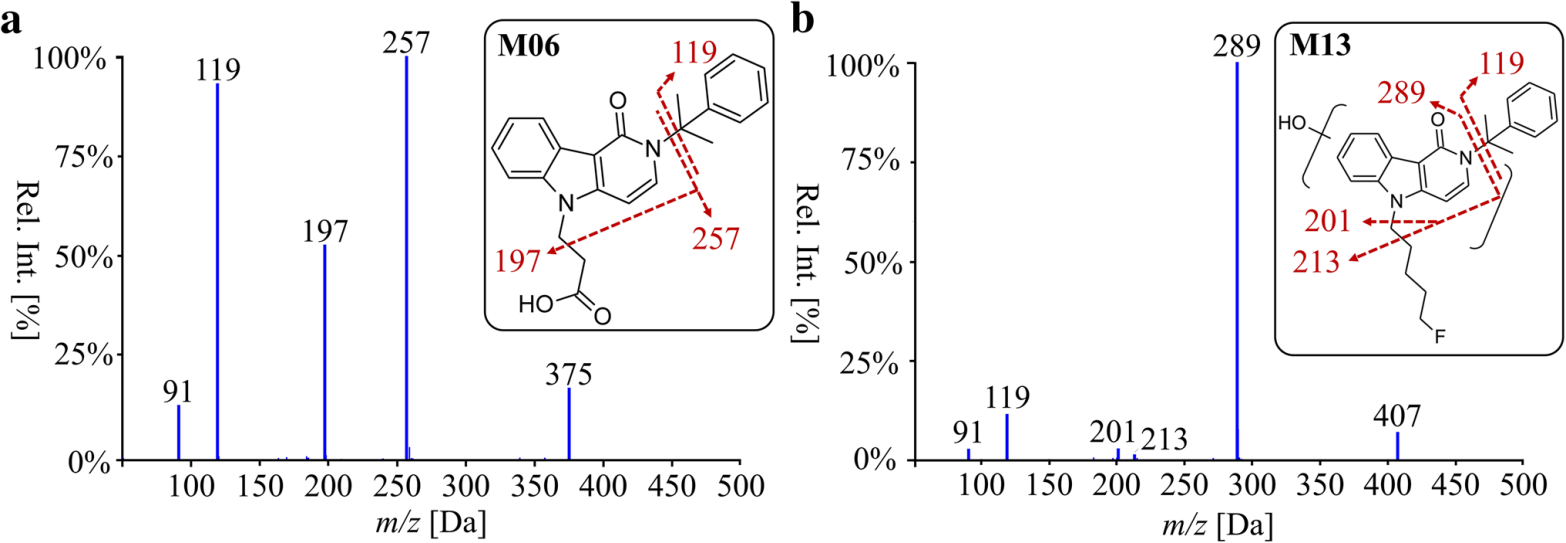
Enhanced product ion spectra by liquid chromatography–tandem mass spectrometry of the main human phase I metabolites **a** M06 and **b** M13. Scans were performed using the optimized declustering potential at 90 V, entrance potential at 10 V and collision energy at 35 V with a spread of ± 15 V

The detection of M13 (overall rank position 3), monohydroxylated at the *γ*-carbolinone core with an unaltered 5-fluoropentyl chain, can facilitate a selective detection of 5F-CUMYL-PEGACLONE uptake in urine screening methods. Figure 4 shows the LC–MS/MS EPI spectra of the suggested analytical targets for urine analysis (M06 and M13).

The product of hydrolytic defluorination M10 (rank position 12) was of relatively low abundance in the analyzed set of urine samples. However, M10 was among the major in vitro phase I metabolites (Table S3) and had been tentatively added to the screening method. It was described for several other SCs that the 5‐hydroxypentyl metabolite was a common main in vitro and in vivo metabolite of SCs with a 5‐fluoropentyl side chain which were prone to hydrolytic defluorination. [12, 23, 24] Surprisingly, this metabolite did not show significant abundances for both of the analogs in human metabolism, which might be explained by quick metabolic oxidation, e.g., to the pentanoic acid metabolite M08 and the propionoic acid metabolite M06.

It should be noted that limitations of a qualitative metabolite ranking based on relative abundances might be biased by matrix effects or many other individual factors like time distance to drug uptake, route of administration and consumption frequency.

## Conclusions

In the present study, the human phase I metabolism of the recently emerged γ-carbolinone derived SC 5F-CUMYL-PEGACLONE was investigated in a collective of human urine specimens. As it has been shown for the non-fluorinated analog CUMYL-PEGACLONE, the drug is subject to extensive metabolism in humans. Metabolic modifications mainly occurred at the *γ*-carbolinone core and the 5-fluoropentyl chain. The main in vivo metabolite M06 is an interesting marker for urine analysis when maximum sensitivity is needed (e.g., drug abstinence testing), but this metabolite can also arise from consumption of the non-fluorinated analog CUMYL-PEGACLONE. With the detection of M06 as the main in vivo metabolite, it was shown that untargeted screening approaches are a suitable tool to detect unexpected biotransformation products enhancing the HLM approach. For reliable differentiation between a consumption of both analogs, the metabolite M13 is suggested as a 5F-CUMYL-PEGACLONE-specific marker. The suggested marker will allow clinical and forensic toxicologists to specifically prove drug uptake by analysis of urine samples. Although the main metabolite was not generated in the HLM assay, the tentatively implemented microsomal metabolites led to the detection of 20 positive urine samples in routine screening, which were used for further evaluation of the most suitable marker metabolites in urine. This points out that HLM assays offer a practical alternative to other models such as human hepatocytes or fungi in order to generate reference spectra of phase I metabolites. Furthermore, the proposed fragmentation patterns and metabolic pathways might facilitate the detection of other *γ*-carbolinone derivatives which might emerge in the near future.

## Electronic supplementary material

Below is the link to the electronic supplementary material.

Supplementary material 1 (DOCX 1094 kb)
