# Supplementary material for: Human phase I metabolism of the novel synthetic cannabinoid 5F-CUMYL-PEGACLONE
Source: Forensic Toxicol. 2018 Oct 5;37(1):154–63. doi: 10.1007/s11419-018-0447-4 (PMC6315001; doi:10.1007/s11419-018-0447-4)
Supplement: Supplementary file 1 — Supplementary material 1 (DOCX 1094 kb) [file 11419_2018_447_MOESM1_ESM.docx]

**Supplementary Material for the article:**

**“Human phase I metabolism of the novel synthetic cannabinoid 5F‑CUMYL‑PEGACLONE”**

**Journal Name: Forensic Toxicology**

Lukas Mogler^1,2^, Sebastian Halter^1^, Maurice Wilde^1,2^, Florian Franz^1,2^, Volker Auwärter^1,^*

^1^ Institute of Forensic Medicine, Forensic Toxicology, Medical Center – University of Freiburg, Faculty of Medicine, University of Freiburg, Albertstr. 9, 79104 Freiburg, Germany

^2^ Hermann Staudinger Graduate School, University of Freiburg, Hebelstraße 27, 79104 Freiburg, Germany

**Table of contents**

| Table S1 | Optimized triple quadrupole mass spectrometric parameters for 5F‑CUMYL-PEGACLONE |
| --- | --- |
| Table S2 | LC-MS/MS sMRM method for the detection of 5F‑CUMYL‑PEGACLONE metabolites |
| Table S3 | Detected *in vitro* metabolites of 5F-CUMYL-PEGACLONE in human liver microsome samples |
| Table S4 | Enhanced Product Ion (EPI) scan spectra of 5F-CUMYL-PEGACLONE *in vivo* metabolites recorded from a urine sample with *in vitro* reference spectra recorded from a pHLM sample |
| Figure S1 | LC-ESI-QToF-MS spectra of 5F-CUMYL-PEGACLONE reference standard solution |
| Figure S2 | Comparative chromatogram of identical *in vivo* metabolites detected in urine samples after 5F‑CUMYL‑PEGACLONE and CUMYL-PEGACLONE uptake |

***Corresponding Author**

Volker Auwärter

Tel.: +49 761 203 6862

Fax: +49 761 203 6826

E-mail: [volker.auwaerter@uniklinik-freiburg.de](mailto:volker.auwaerter@uniklinik-freiburg.de)

**Table S1** Optimized mass spectrometric parameters of the scheduled multiple reaction monitoring (sMRM) ion transitions of 5F-CUMYL-PEGACLONE in the order of their intensities beginning with the most intensive transition.

| **Q1 Mass [Da]** | **Q3 Mass [Da]** | **DP [V]** | **EP [V]** | **CE [V]** | **CXP [V]** |
| --- | --- | --- | --- | --- | --- |
| 391 | 273 | 90 | 10 | 27 | 23 |
| 391 | 91 | 90 | 10 | 75 | 12 |
| 391 | 167 | 90 | 10 | 69 | 25 |
| 391 | 119 | 90 | 10 | 35 | 20 |
| 391 | 185 | 90 | 10 | 57 | 25 |
| 391 | 197 | 90 | 10 | 61 | 27 |

DP (declustering potential), EP (entrance potential), CE (collision energy), CXP (cell exit potential)

Table S2 Specific mass transitions of the detected metabolites in order of the retention time (RT) included in the sMRM method used for the analysis of urine samples

| ID  in vivo | RT [min] | ID | Q1 | Q3 | DP | EP | CE | CXP |
| --- | --- | --- | --- | --- | --- | --- | --- | --- |
| n.d. | 2.0 | Di-hydroxylation_1 | 423 | 273 | 90 | 10 | 27 | 23 |
|  | 2.0 | Di-hydroxylation _2 | 423 | 185 | 90 | 10 | 57 | 25 |
| n.d. | 2.2 | Di-hydroxylation _1 | 423 | 273 | 90 | 10 | 27 | 23 |
|  | 2.2 | Di-hydroxylation _2 | 423 | 185 | 90 | 10 | 57 | 25 |
| M01 | 2.6 | *N*-Dealkylation +mono-hydroxylation _1 | 319 | 201 | 90 | 10 | 57 | 25 |
|  | 2.6 | *N*-Dealkylation +mono-hydroxylation _2 | 319 | 119 | 90 | 10 | 35 | 20 |
| M02 | 3.1 | *N*-Dealkylation +mono-hydroxylation _1 | 319 | 201 | 90 | 10 | 57 | 25 |
|  | 3.1 | *N*-Dealkylation +mono-hydroxylation _2 | 319 | 119 | 90 | 10 | 35 | 20 |
| n.d. | 3.2 | Hydrolytic defluorination+mono-hydroxylation _1 | 405 | 271 | 90 | 10 | 27 | 23 |
|  | 3.2 | Hydrolytic defluorination+mono-hydroxylation _2 | 405 | 135 | 90 | 10 | 35 | 20 |
| n.d. | 3.3 | Hydrolytic defluorination+mono-hydroxylation _1 | 405 | 287 | 90 | 10 | 27 | 23 |
|  | 3.3 | Hydrolytic defluorination+mono-hydroxylation _2 | 405 | 197 | 90 | 10 | 61 | 27 |
| n.d. | 3.5 | Hydrolytic defluorination+mono-hydroxylation _1 | 405 | 271 | 90 | 10 | 27 | 23 |
|  | 3.5 | Hydrolytic defluorination+mono-hydroxylation _1 | 405 | 135 | 90 | 10 | 35 | 20 |
| M03 | 3.5 | Di-hydroxylation _1 | 423 | 305 | 90 | 10 | 27 | 23 |
|  | 3.5 | Di-hydroxylation _2 | 423 | 201 | 90 | 10 | 57 | 25 |
| n.d. | 3.6 | Hydrolytic defluorination+mono-hydroxylation _1 | 405 | 287 | 90 | 10 | 27 | 23 |
|  | 3.6 | Hydrolytic defluorination+mono-hydroxylation _1 | 405 | 201 | 90 | 10 | 57 | 25 |
| M04 | 3.8 | Hydrolytic defluorination+mono-hydroxylation _1 | 405 | 271 | 90 | 10 | 27 | 23 |
|  | 3.8 | Hydrolytic defluorination+mono-hydroxylation _2 | 405 | 135 | 90 | 10 | 35 | 20 |
| M05 | 3.8 | Di-hydroxylation _1 | 423 | 305 | 90 | 10 | 27 | 23 |
|  | 3.8 | Di-hydroxylation _2 | 423 | 213 | 90 | 10 | 61 | 27 |
| M06 | 4.4 | Propionic acid_1 | 375 | 257 | 90 | 10 | 27 | 23 |
|  | 4.4 | Propionic acid_2 | 375 | 119 | 90 | 10 | 47 | 13 |
| n.d. | 4.4 | Hydrolytic defluorination+mono-hydroxylation _1 | 405 | 271 | 90 | 10 | 27 | 23 |
|  | 4.4 | Hydrolytic defluorination+mono-hydroxylation _2 | 405 | 135 | 90 | 10 | 35 | 20 |
| n.d. | 4.5 | Ketone formation+mono-hydroxylation_1 | 403 | 285 | 90 | 10 | 27 | 23 |
|  | 4.5 | Ketone formation+mono-hydroxylation_2 | 403 | 197 | 90 | 10 | 61 | 27 |
| M07 | 4.8 | Hydrolytic defluorination+mono-hydroxylation _1 | 405 | 287 | 90 | 10 | 27 | 23 |
|  | 4.8 | Hydrolytic defluorination+mono-hydroxylation _2 | 405 | 201 | 90 | 10 | 57 | 25 |
| n.d. | 4.9 | *N*-Dealkylation _1 | 303 | 185 | 90 | 10 | 57 | 25 |
|  | 4.9 | *N*-Dealkylation _2 | 303 | 167 | 90 | 10 | 69 | 25 |
| n.d. | 5.6 | Di-hydroxylation _1 | 423 | 273 | 90 | 10 | 27 | 23 |
|  | 5.6 | Di-hydroxylation _2 | 423 | 185 | 90 | 10 | 57 | 25 |
| M08 | 5.9 | Pentanoic acid_1 | 403 | 285 | 90 | 10 | 27 | 23 |
|  | 5.9 | Pentanoic acid_2 | 403 | 185 | 90 | 10 | 57 | 25 |
| M09 | 6.0 | Dihydrodiol formation_1 | 425 | 307 | 90 | 10 | 27 | 23 |
|  | 6.0 | Dihydrodiol formation_2 | 425 | 119 | 90 | 10 | 35 | 20 |
| n.d. | 6.1 | Mono-hydroxylation _1 | 407 | 289 | 90 | 10 | 27 | 23 |
|  | 6.1 | Mono-hydroxylation _2 | 407 | 197 | 90 | 10 | 61 | 27 |
| M10 | 6.3 | Hydrolytic defluorination_1 | 389 | 271 | 90 | 10 | 27 | 23 |
|  | 6.3 | Hydrolytic defluorination_2 | 389 | 197 | 90 | 10 | 61 | 27 |
| M11 | 6.5 | Mono-hydroxylation _1 | 407 | 289 | 90 | 10 | 27 | 23 |
|  | 6.5 | Mono-hydroxylation _2 | 407 | 197 | 90 | 10 | 61 | 27 |
| n.d. | 7.1 | Mono-hydroxylation _1 | 407 | 289 | 90 | 10 | 27 | 23 |
|  | 7.1 | Mono-hydroxylation _2 | 407 | 197 | 90 | 10 | 61 | 27 |
| M12 | 7.8 | Mono-hydroxylation _1 | 407 | 289 | 90 | 10 | 27 | 23 |
|  | 7.8 | Mono-hydroxylation _2 | 407 | 201 | 90 | 10 | 57 | 25 |
| n.d. | 8.0 | Ketone formation _1 | 405 | 287 | 90 | 10 | 27 | 23 |
|  | 8.0 | Ketone formation _2 | 405 | 197 | 90 | 10 | 61 | 27 |
| n.d. | 8.2 | Mono-hydroxylation _1 | 407 | 273 | 90 | 10 | 27 | 23 |
|  | 8.2 | Mono-hydroxylation _2 | 407 | 135 | 90 | 10 | 35 | 20 |
| n.d. | 8.5 | Mono-hydroxylation _1 | 407 | 289 | 90 | 10 | 27 | 23 |
|  | 8.5 | Mono-hydroxylation _2 | 407 | 201 | 90 | 10 | 57 | 25 |
| M13 | 8.6 | Mono-hydroxylation _1 | 407 | 273 | 90 | 10 | 27 | 23 |
|  | 8.6 | Mono-hydroxylation _2 | 407 | 135 | 90 | 10 | 35 | 20 |
| n.d. | 9.3 | Mono-hydroxylation _1 | 407 | 273 | 90 | 10 | 27 | 23 |
|  | 9.3 | Mono-hydroxylation _2 | 407 | 135 | 90 | 10 | 35 | 20 |
| M14 | 9.6 | Mono-hydroxylation _1 | 407 | 289 | 90 | 10 | 27 | 23 |
|  | 9.6 | Mono-hydroxylation _2 | 407 | 201 | 90 | 10 | 57 | 25 |
| n.d. | 10.1 | Ketone formation _1 | 405 | 273 | 90 | 10 | 27 | 23 |
|  | 10.1 | Ketone formation _2 | 405 | 197 | 90 | 10 | 61 | 27 |
| n.d. | 10.7 | 5F-CUMYL-PEGACLONE 1 | 391 | 273 | 90 | 10 | 27 | 23 |
|  | 10.7 | 5F-CUMYL-PEGACLONE 2 | 391 | 119 | 90 | 10 | 35 | 20 |

*n.d* not detectable in the authentic urine samples

Table S3 Detected in vitro metabolites of 5F-CUMYL-PEGACLONE in pooled human liver microsome (pHLM) samples (triplicates) in the order of their retention times (RT)

| **ID** | **RT [min]** | **Compound Label** | **Location** | **Mean area [cps]** | **Meas. *m/z*** | **Ion formula** | ***m/z*** | **Error [ppm]** |
| --- | --- | --- | --- | --- | --- | --- | --- | --- |
| - | 2.0 | Di-hydroxylation | CUM | 1.43E+06 | 423.2079 | C_25_H_28_FN_2_O_3_ | 423.2078 | -0.1 |
| - | 2.2 | Di-hydroxylation | CUM | 3.93E+05 | 423.2079 | C_25_H_28_FN_2_O_3_ | 423.2078 | -0.2 |
| - | 3.2 | Hydrolytic defluorination + mono-hydroxylation | CUM | 6.12E+05 | 405.2174 | C_25_H_29_N_2_O_3_ | 405.2173 | -0.4 |
| - | 3.3 | Hydrolytic defluorination + mono-hydroxylation | 5F-P | 2.88E+05 | 405.2175 | C_25_H_29_N_2_O_3_ | 405.2173 | -0.6 |
| - | 3.5 | Hydrolytic defluorination + mono-hydroxylation | CUM | 2.43E+05 | 405.2175 | C_25_H_29_N_2_O_3_ | 405.2173 | -0.6 |
| M03 | 3.5 | Di-hydroxylation | CBL, 5F-P | 2.60E+05 | 423.2081 | C_25_H_28_FN_2_O_3_ | 423.2078 | -0.6 |
| M04 | 3.6 | Hydrolytic defluorination + mono-hydroxylation | CBL | 1.24E+06 | 405.2173 | C_25_H_29_N_2_O_3_ | 405.2173 | -0.1 |
| - | 3.8 | Hydrolytic defluorination + mono-hydroxylation | CUM | 5.40E+05 | 405.2175 | C_25_H_29_N_2_O_3_ | 405.2173 | -0.5 |
| M05 | 3.8 | Di-hydroxylation | CBL, 5F-P | 2.95E+05 | 423.2082 | C_25_H_28_FN_2_O_3_ | 423.2078 | -0.9 |
| - | 4.4 | Hydrolytic defluorination + mono-hydroxylation | CUM | 1.68E+05 | 405.2178 | C_25_H_29_N_2_O_3_ | 405.2173 | -1.2 |
| - | 4.5 | Ketone formation + mono-hydroxylation | 5F-P | 3.53E+05 | 403.2017 | C_25_H_27_N_2_O_3_ | 403.2016 | -0.2 |
| M07 | 4.7 | Hydrolytic defluorination + mono-hydroxylation | CBL | 2.33E+05 | 405.2173 | C_25_H_29_N_2_O_3_ | 405.2173 | -0.1 |
| - | 4.9 | *N*-Dealkylation | 5F-P | 6.51E+05 | 303.1491 | C_20_H_19_N_2_O | 303.1492 | 0.3 |
| - | 5.6 | Di-hydroxylation | CUM | 2.69E+05 | 423.208 | C_25_H_28_FN_2_O_3_ | 423.2078 | -0.3 |
| M08 | 6.0 | Pentanoic acid | 5F-P | 4.26E+06 | 403.2017 | C_25_H_27_N_2_O_3_ | 403.2016 | -0.1 |
| M09 | 6.0 | Dihydrodiol formation | CBL | 3.12E+05 | 407.2132 | C_25_H_28_FN_2_O_2_ | 407.2129 | -0.6 |
| - | 6.1 | Mono-hydroxylation | 5F-P | 5.48E+06 | 407.2132 | C_25_H_28_FN_2_O_2_ | 407.2129 | -0.6 |
| M10 | 6.3 | Hydrolytic defluorination | 5F-P | 2.37E+07 | 389.2222 | C_25_H_29_N_2_O_2_ | 389.2224 | 0.5 |
| M11 | 6.5 | Mono-hydroxylation | 5F-P | 8.38E+06 | 407.2129 | C_25_H_28_FN_2_O_2_ | 407.2129 | 0.1 |
| M12 | 7.1 | Mono-hydroxylation | 5F-P | 1.30E+07 | 407.2131 | C_25_H_28_FN_2_O_2_ | 407.2129 | -0.4 |
| M13 | 7.8 | Mono-hydroxylation | CBL | 9.16E+06 | 407.2132 | C_25_H_28_FN_2_O_2_ | 407.2129 | -0.8 |
| - | 8.0 | Ketone formation | 5F-P | 3.80E+06 | 405.1975 | C_25_H_26_FN_2_O_2_ | 405.1973 | -0.6 |
| - | 8.2 | Mono-hydroxylation | CUM | 9.23E+05 | 407.2129 | C_25_H_28_FN_2_O_2_ | 407.2129 | 0.0 |
| - | 8.5 | Mono-hydroxylation | CBL | 4.41E+05 | 407.2131 | C_25_H_28_FN_2_O_2_ | 407.2129 | -0.4 |
| M14 | 8.6 | Mono-hydroxylation | CUM | 1.38E+06 | 407.2131 | C_25_H_28_FN_2_O_2_ | 407.2129 | -0.3 |
| - | 9.3 | Mono-hydroxylation | CUM | 8.50E+05 | 407.2133 | C_25_H_28_FN_2_O_2_ | 407.2129 | -0.8 |
| M15 | 9.6 | Mono-hydroxylation | CBL | 1.87E+06 | 407.2133 | C_25_H_28_FN_2_O_2_ | 407.2129 | -1.0 |
| - | 10.1 | Ketone formation | CUM | 5.30E+05 | 405.1977 | C_25_H_26_FN_2_O_2_ | 405.1973 | -1.1 |
| - | 10.7 | Parent compound | n.a. | 5.10E+07 | 391.2182 | C_25_H_28_FN_2_O | 391.2182 | -0.5 |

The mean area is given for each metabolite detected in the three pHLM assays samples for the most abundant MRM ion transition. CUM=Cumy-moiety, 5F-P=5-fluoropentyl chain, CBL=*γ-*carbolinone core system

Table S4 Enhanced product ion (EPI) spectra of 5F-CUMYL-PEGACLONE metabolites detected in authentic urine samples after treatment with *β*-glucuronidase compared to corresponding signals in pooled human liver microsome (pHLM) samples.

| Metabolite | LC–MS/MS EPI spectra from in vivo sample (urine) | LC–MS/MS EPI spectra from in vitro sample (pHLM) |
| --- | --- | --- |
| 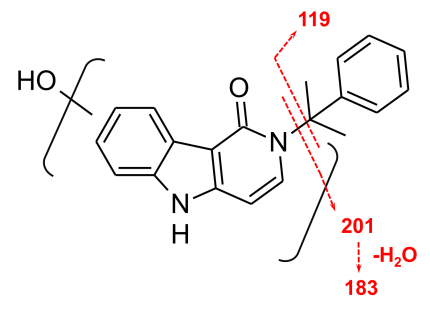M01  RT: 2.6 min  [M+H]^+^: 319.1441  Ion formula: C_20_H_19_N_2_O_2_ | 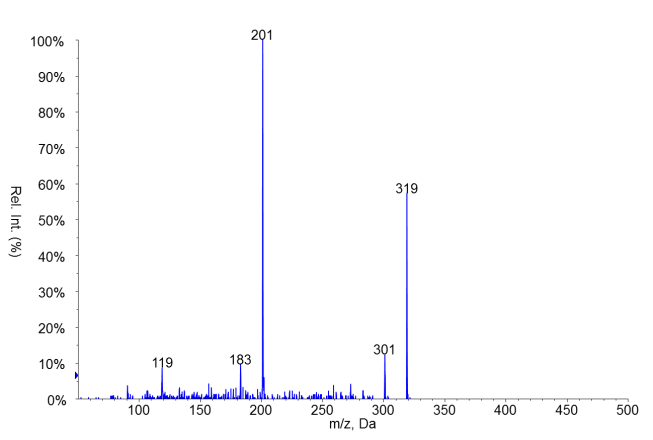 | Not detected in pHLM assay |
| 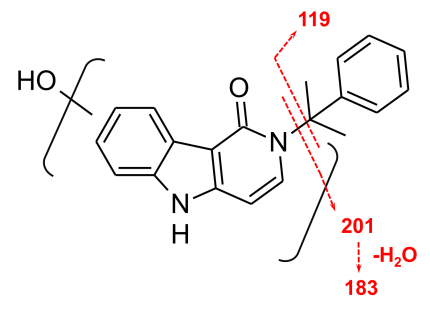M02  RT: 3.1 min  [M+H]^+^: 319.1441  Ion formula: C_20_H_19_N_2_O_2_ | 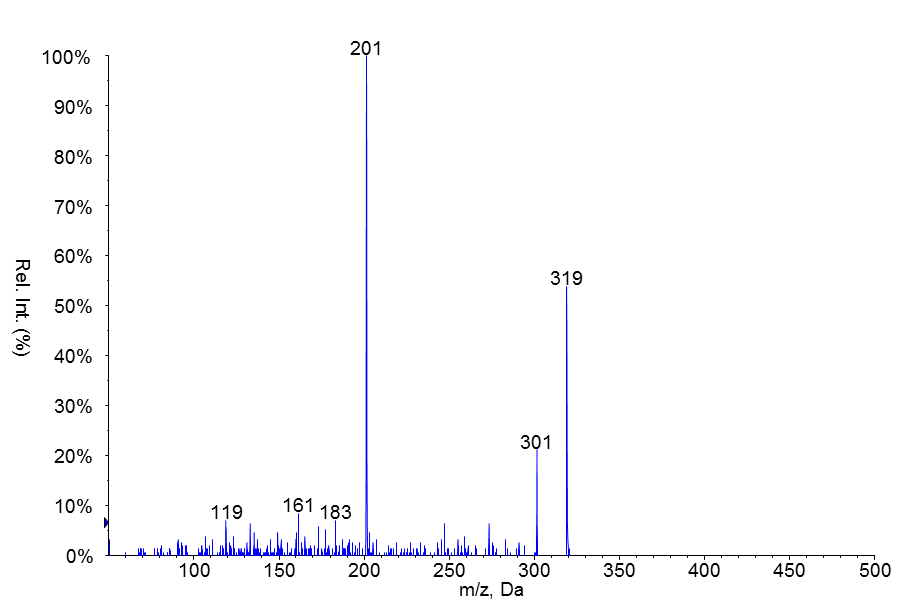 | Not detected in pHLM assay |
| 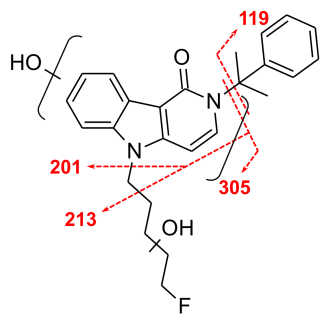  M03  RT: 3.5 min  [M+H]^+^: 423.2078  Ion formula: C_25_H_28_FN_2_O_3_ | 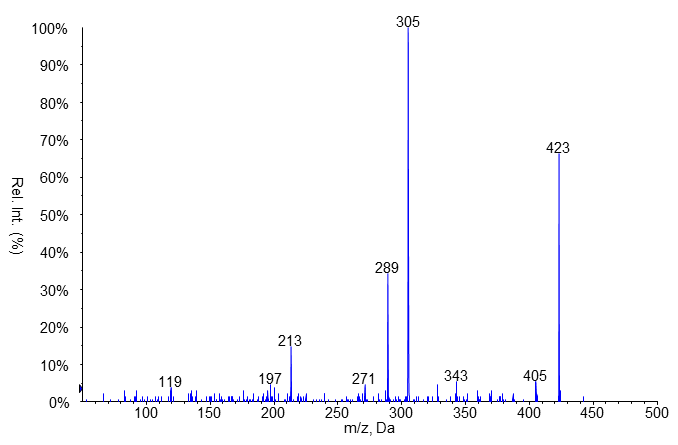 | 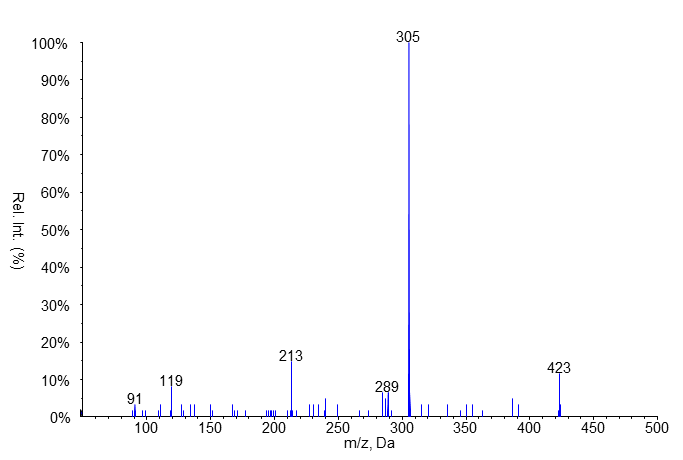 |
| 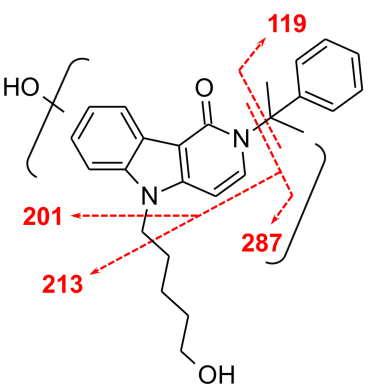  M04  RT: 3.6 min  [M+H]^+^: 405.2173  Ion formula: C_25_H_29_N_2_O_3_ | 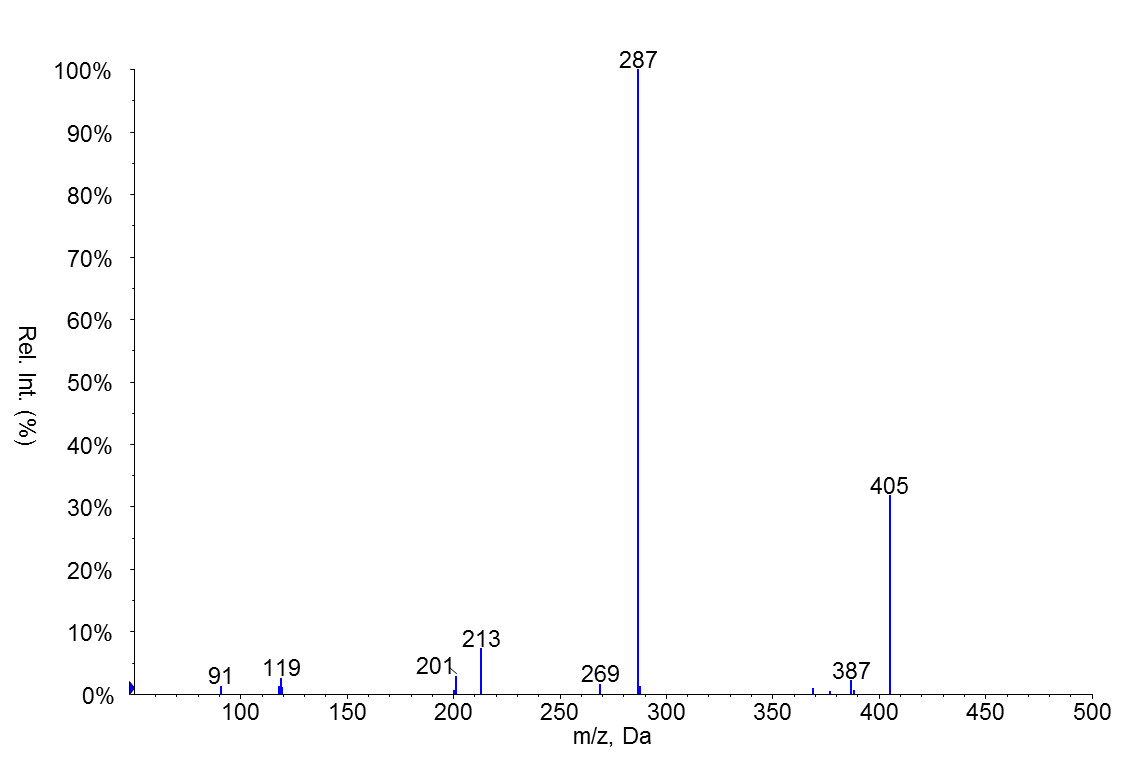 | 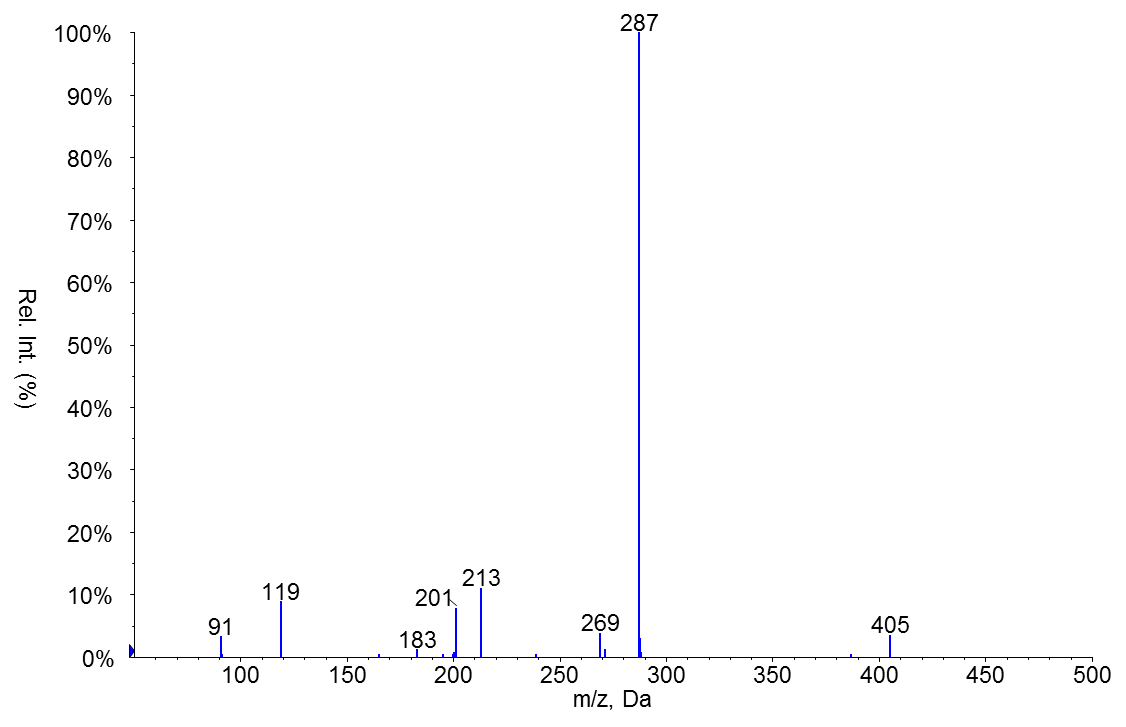 |
| 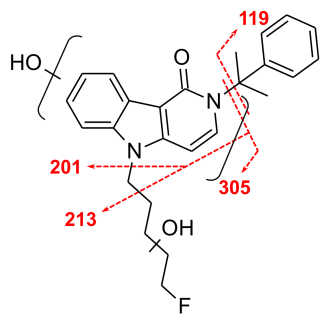  M05  RT: 3.8 min  [M+H]^+^: 423.2078  Ion formula: C_25_H_28_FN_2_O_3_ | 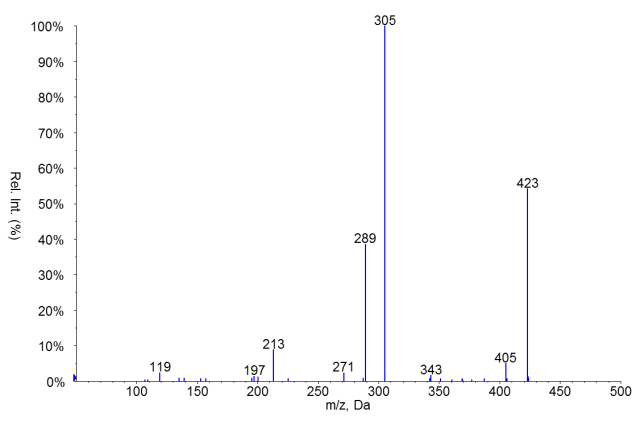 | 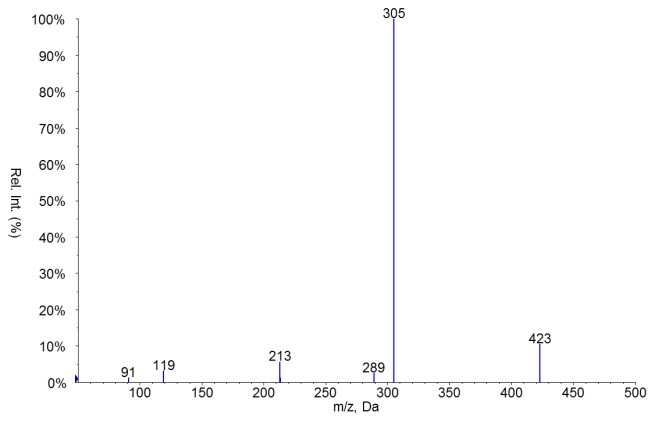 |
| 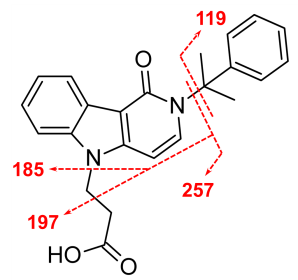  M06  RT: 4.4 min  [M+H]^+^: 375.1703  Ion formula: C_23_H_23_N_2_O_3_ | 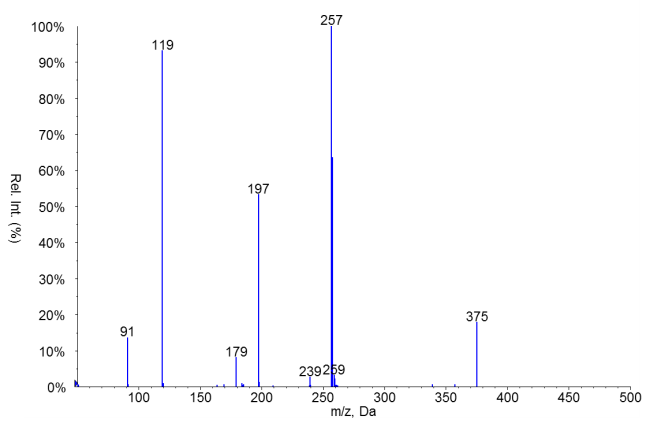 | Not detected in pHLM assay |
| 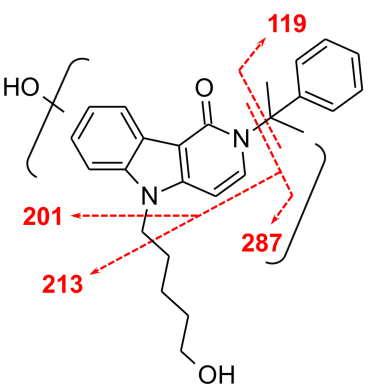  M07  RT: 4.7 min  [M+H]^+^: 405.2173  Ion formula: C_25_H_29_N_2_O_3_ | 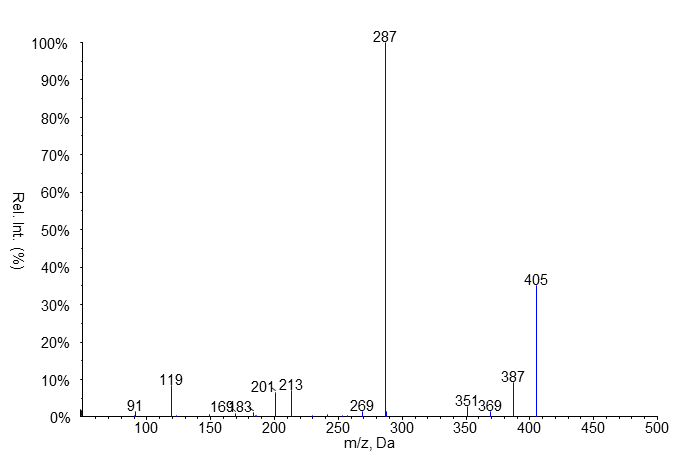 | 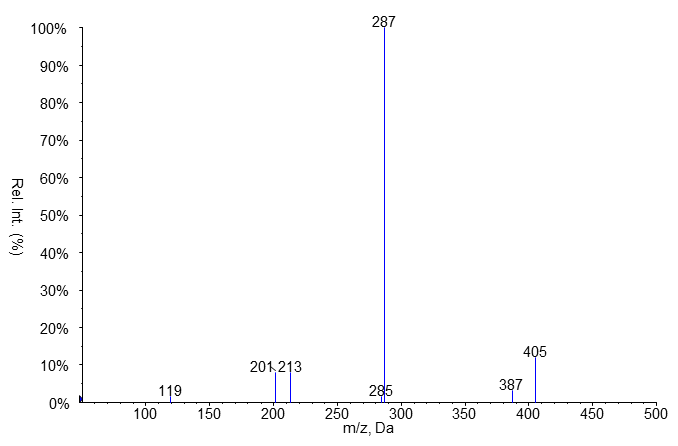 |
| 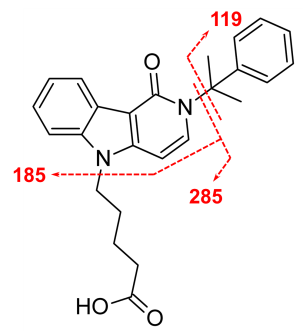  M08  RT: 6.0 min  [M+H]^+^: 403.2016  Ion formula: C_25_H_27_N_2_O_3_ | 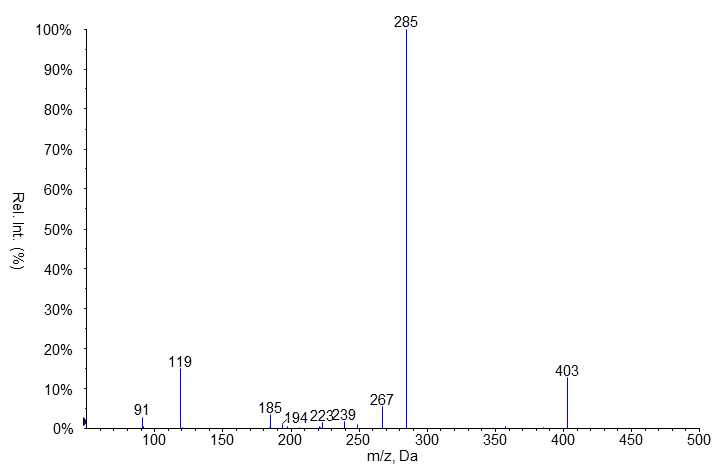 | 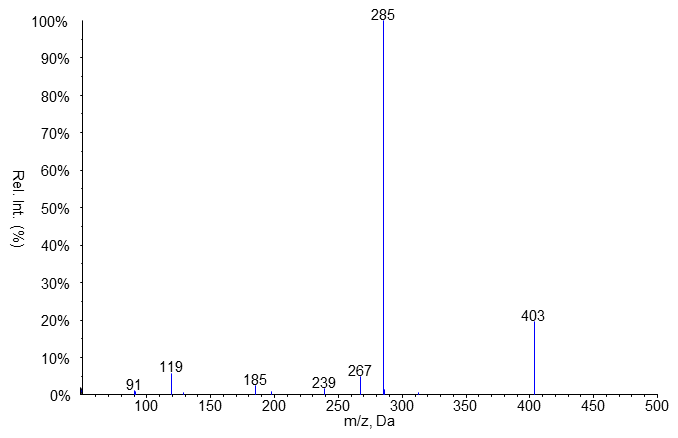 |
| 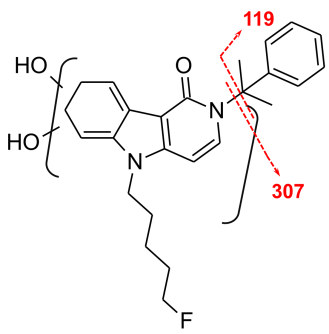  M09  RT: 6.0 min  [M+H]^+^: 425.2235  Ion formula: C_25_H_30_FN_2_O_3_ | 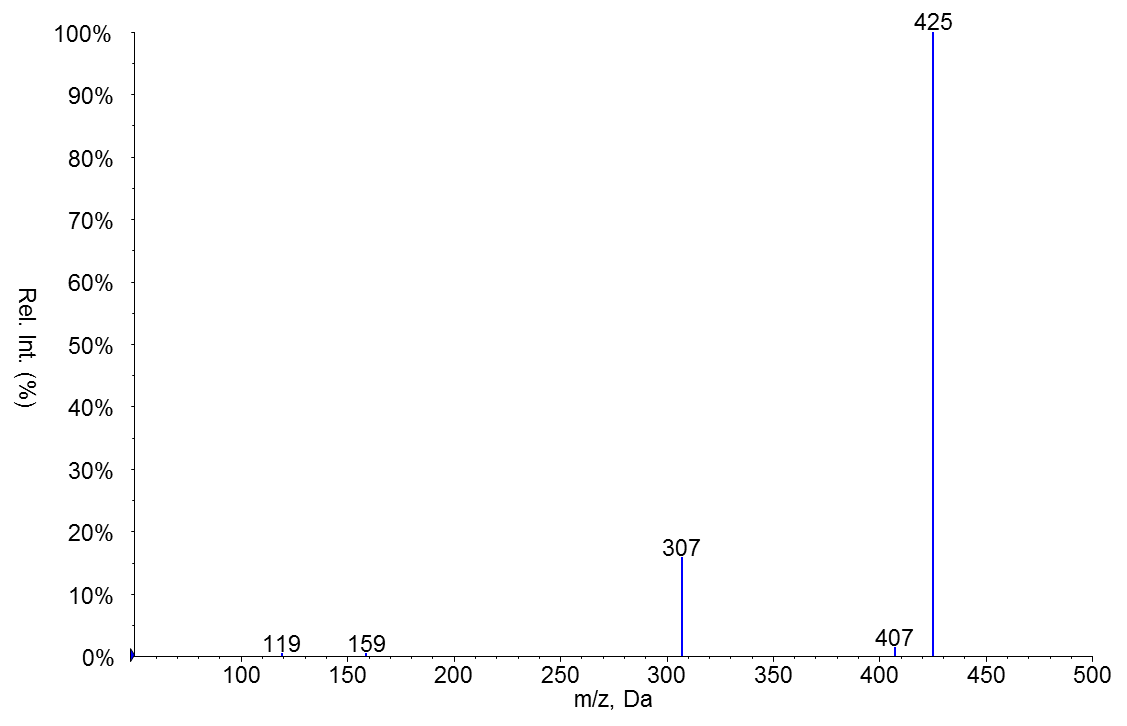 | 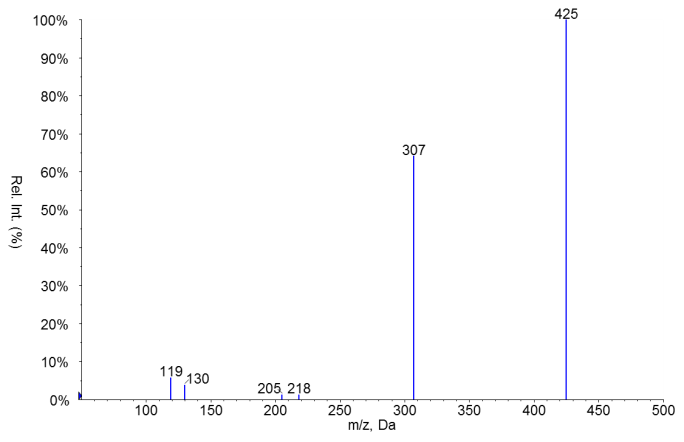 |
| 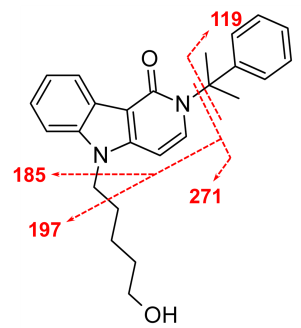  M10  RT: 6.3 min  [M+H]^+^: 389.2224  Ion formula: C_25_H_29_N_2_O_2_ | 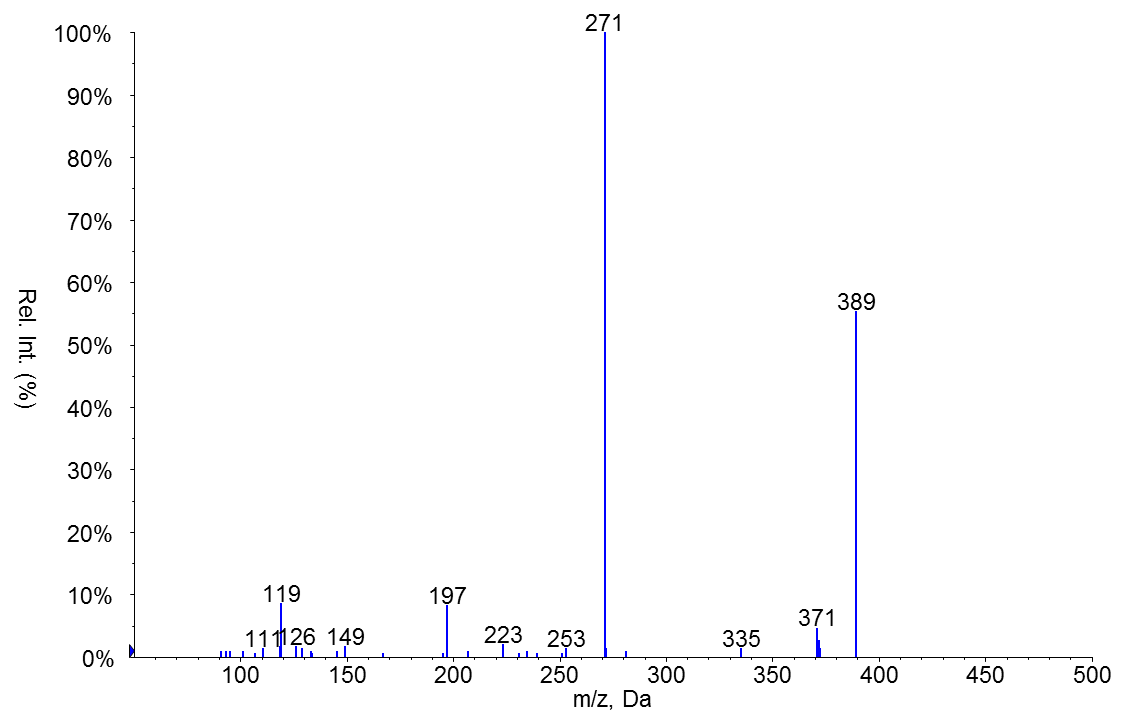 | 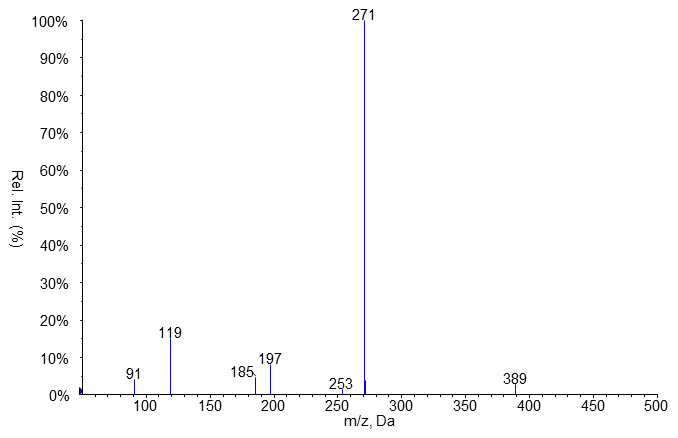 |
| 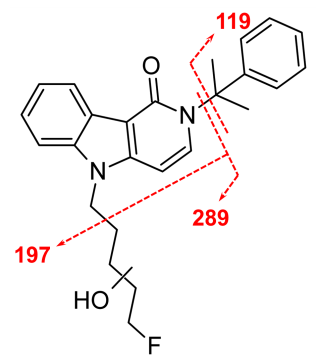  M11  RT: 6.5 min  [M+H]^+^: 407.2129  Ion formula: C_25_H_28_FN_2_O_2_ | 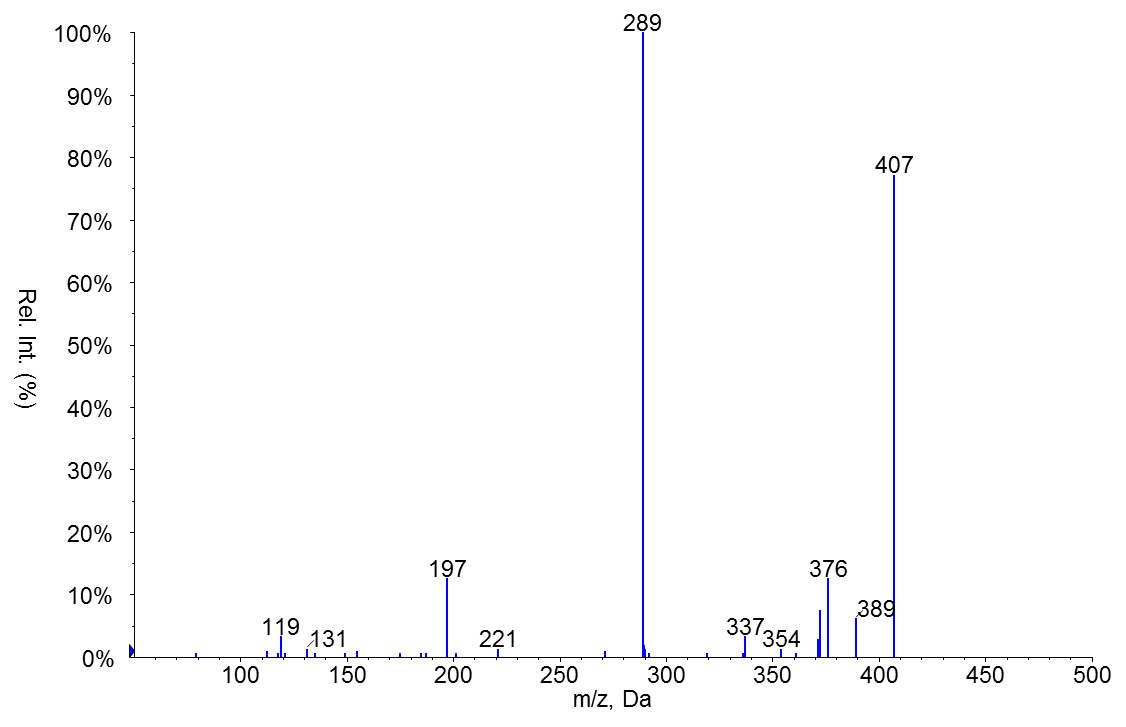 | 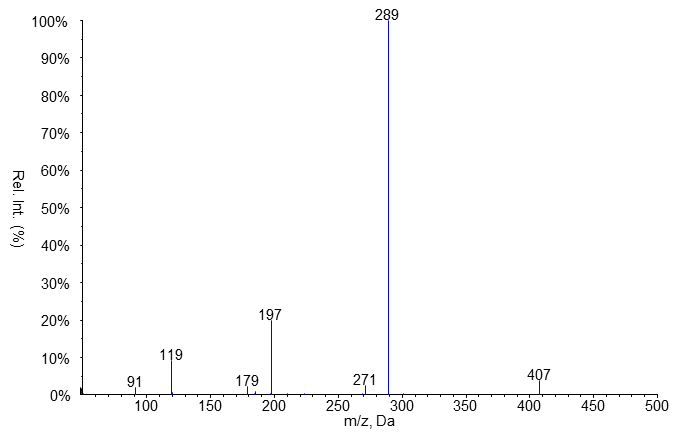 |
| 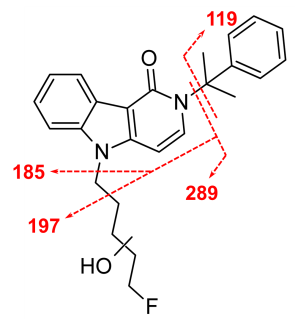  M12  RT: 7.1 min  [M+H]^+^: 407.2129  Ion formula: C_25_H_28_FN_2_O_2_ | 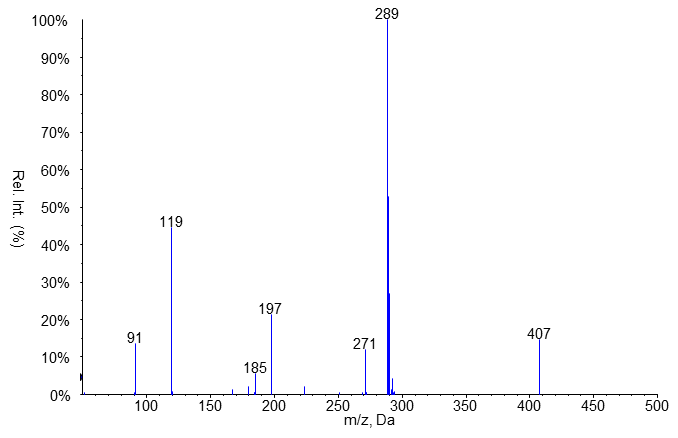 | 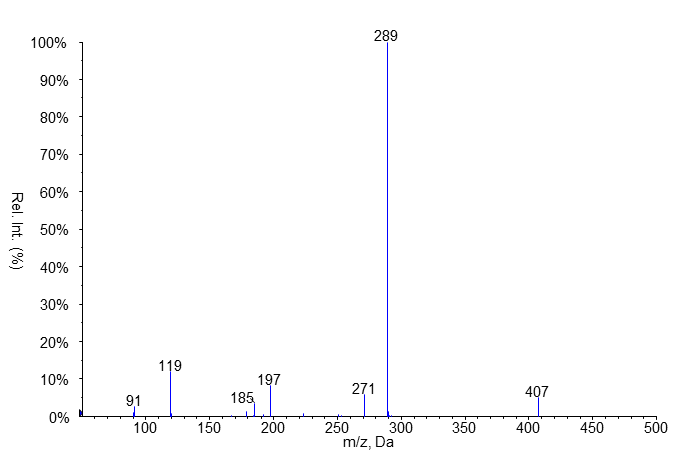 |
| 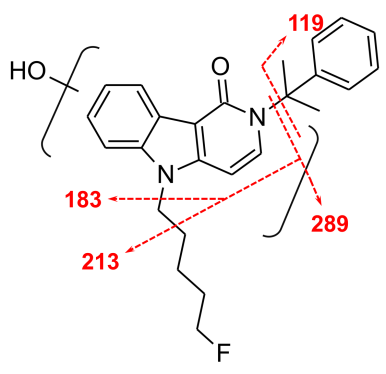  M13  RT: 7.8 min  [M+H]^+^: 407.2129  Ion formula: C_25_H_28_FN_2_O_2_ | 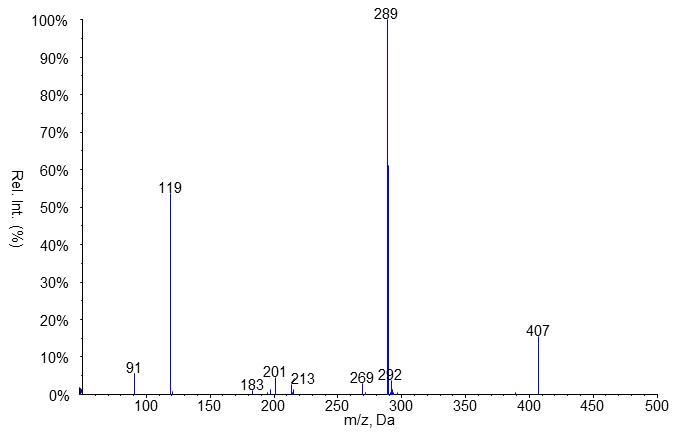 | 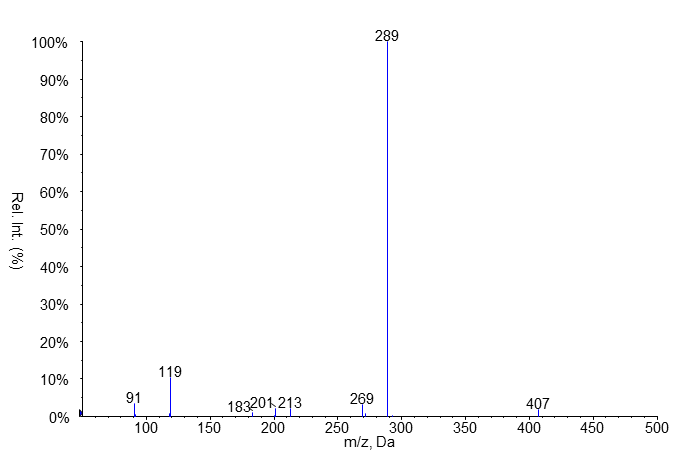 |
| 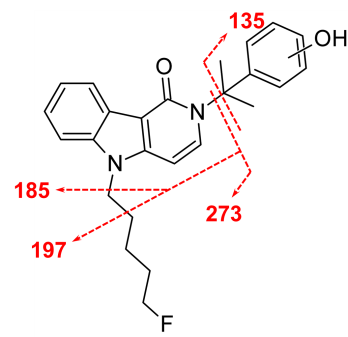  M14  RT: 8.6 min  [M+H]^+^: 407.2129  Ion formula: C_25_H_28_FN_2_O_2_ | 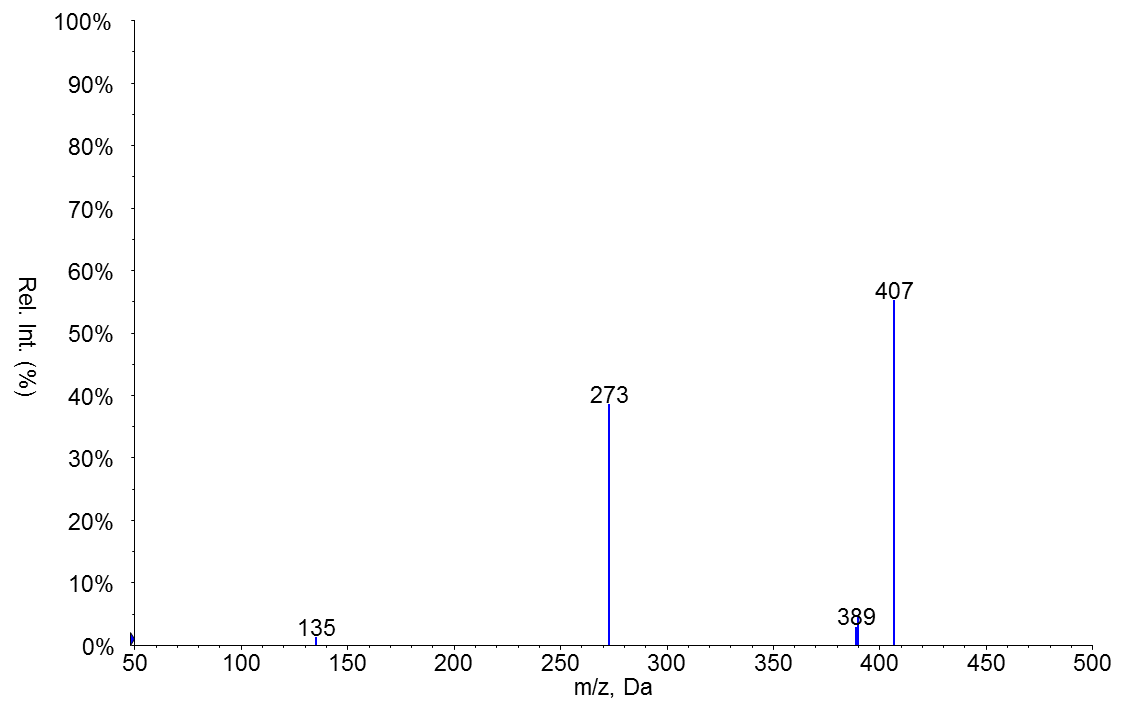 | 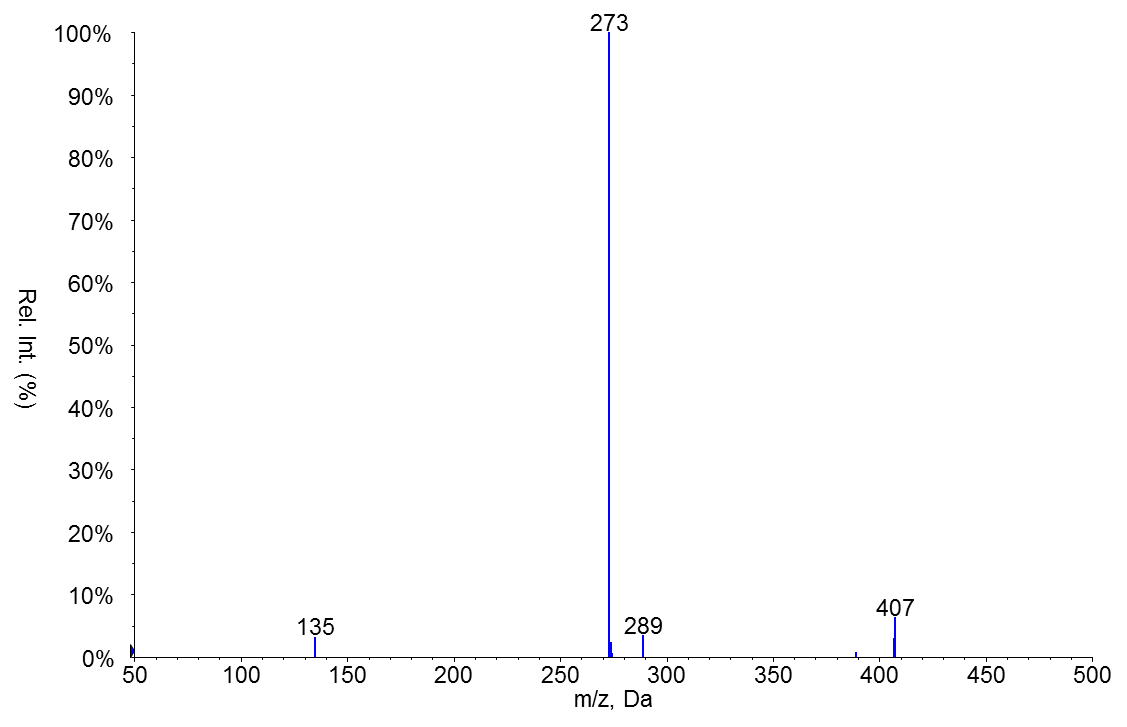 |
| 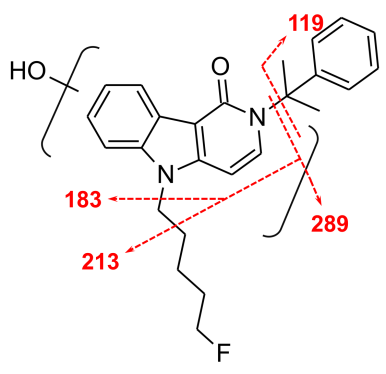  M15  RT: 9.6 min  [M+H]^+^: 407.2129  Ion formula: C_25_H_28_FN_2_O_2_ | 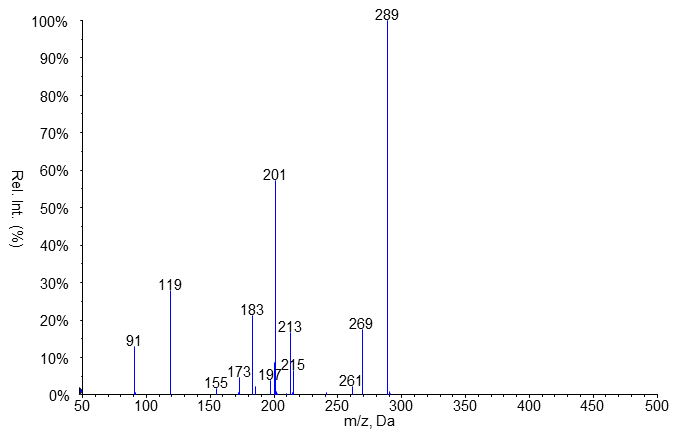 | 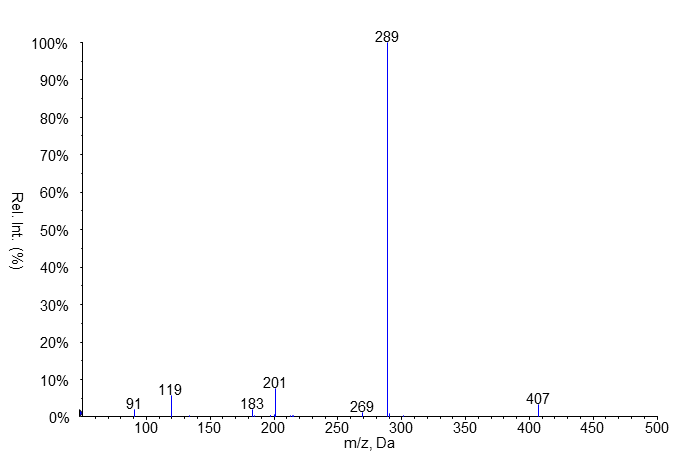 |

Proposed structural formulae and fragmentation are shown for each compound along with their molecular formulae, single-protonated masses and retention times (RT). The EPI scans were recorded with a declustering potential of 90 V, entrance potential of 10 V and collision energy of 35 V with a collision energy spread of ± 15 V


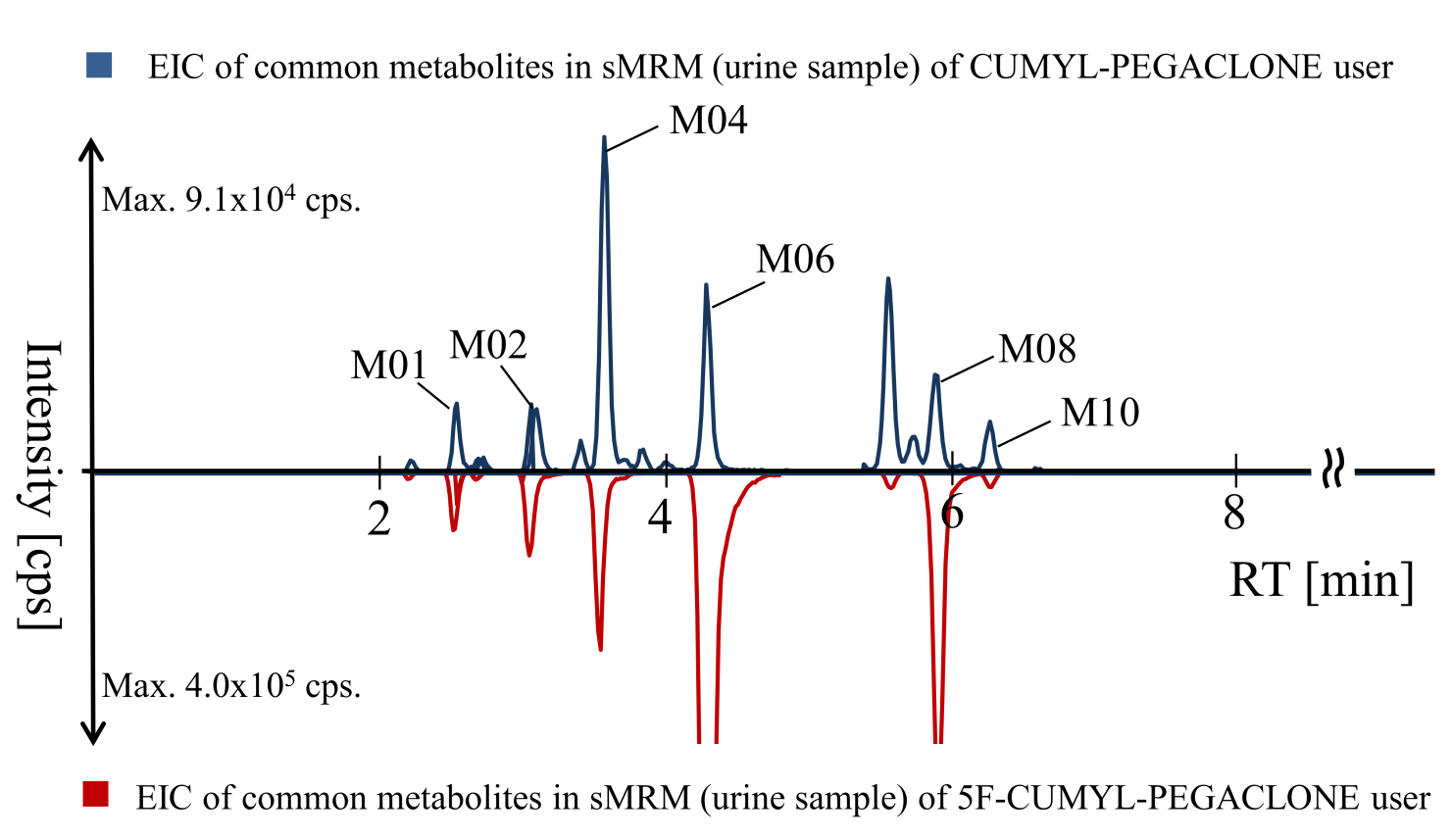

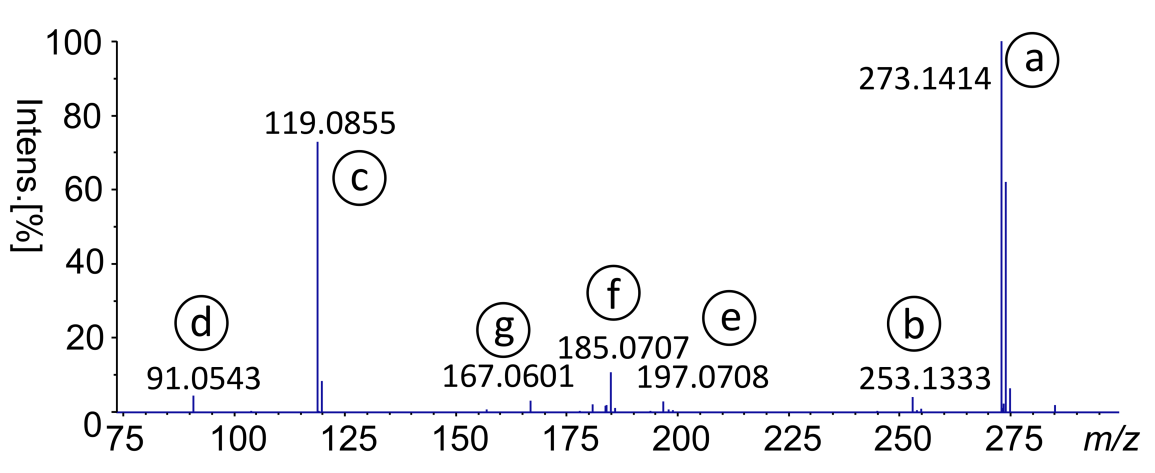


Fig. S2 Comparative extracted ion chromatograms (sMRM scan see Supplemental Material Table S2) of common metabolites of Cumyl-PEGACLONE (blue) and 5F-Cumyl-PEGACLONE (red) recorded from urine samples of drug users.

Fig. S1 LC–ESI-QToF-MS spectra for the investigation of the collision induced dissociation (CID) pathways recorded from a 1 µg/mL 5F-CUMYL-PEGACLONE solution
